# Evolution of the Genes Encoding Effector Candidates Within Multiple Pathotypes of *Magnaporthe oryzae*

**DOI:** 10.3389/fmicb.2019.02575

**Published:** 2019-11-06

**Authors:** Ki-Tae Kim, Jaeho Ko, Hyeunjeong Song, Gobong Choi, Hyunbin Kim, Jongbum Jeon, Kyeongchae Cheong, Seogchan Kang, Yong-Hwan Lee

**Affiliations:** ^1^Department of Agricultural Biotechnology, Seoul National University, Seoul, South Korea; ^2^Interdisciplinary Program in Agricultural Genomics, Seoul National University, Seoul, South Korea; ^3^Department of Plant Pathology and Environmental Microbiology, The Pennsylvania State University, State College, PA, United States; ^4^Center for Fungal Genetic Resources, Seoul National University, Seoul, South Korea; ^5^Plant Immunity Research Center, Seoul National University, Seoul, South Korea; ^6^Research Institute of Agriculture and Life Sciences, Seoul National University, Seoul, South Korea

**Keywords:** avirulence, comparative genomics, effector, host specialization, *M. grisea*, *M. oryzae*

## Abstract

*Magnaporthe oryzae* infects rice, wheat, and many grass species in the Poaceae family by secreting protein effectors. Here, we analyzed the distribution, sequence variation, and genomic context of effector candidate (*EFC*) genes in 31 isolates that represent five pathotypes of *M. oryzae*, three isolates of *M. grisea*, a sister species of *M. oryzae*, and one strain each for eight species in the family Magnaporthaceae to investigate how the host range expansion of *M. oryzae* has likely affected the evolution of effectors. We used the *EFC* genes of *M. oryzae* strain 70-15, whose genome has served as a reference for many comparative genomics analyses, to identify their homologs in these strains. We also analyzed the previously characterized avirulence (*AVR*) genes and single-copy orthologous (*SCO*) genes in these strains, which showed that the *EFC* and *AVR* genes evolved faster than the *SCO* genes. The *EFC* and *AVR* repertoires among *M. oryzae* pathotypes varied widely probably because adaptation to individual hosts exerted different types of selection pressure. Repetitive DNA elements appeared to have caused the variation of some *EFC* genes. Lastly, we analyzed expression patterns of the *AVR* and *EFC* genes to test the hypothesis that such genes are preferentially expressed during host infection. This comprehensive dataset serves as a foundation for future studies on the genetic basis of the evolution and host specialization in *M. oryzae*.

## Introduction

Like other groups of plant pathogens, fungal pathogens secrete diverse effector proteins to manipulate host defense signaling pathways and downstream machinery. Although most effectors function to enhance virulence (Greenshields and Jones, 2008), some effectors trigger strong defense responses in host varieties that express specific resistance (*R*) gene products, and such effectors are categorized as avirulence (AVR) effectors (Zhang and Xu, 2014). The effector repertoire of each strain, including both the virulence and AVR effectors, determines its host specialization (Sánchez-Vallet et al., 2018). Due to the ‘arms race’ between plants and pathogens, effector genes and host resistance genes rapidly coevolve to overcome host defense and pathogen attack, respectively (Anderson et al., 2010). Accordingly, pathogens likely need to change their effector repertoire to infect a new host. Such changes include the acquisition of new effector gene(s), optimization of existing effectors for the new host, and modification of specific *AVR* genes in ways to avoid host recognition (Sánchez-Vallet et al., 2018). A comprehensive understanding of how such evolutionary changes have occurred and underlying mechanisms are crucial to help develop and deploy effective disease control measures (Dodds et al., 2009; Sánchez-Vallet et al., 2018). In this study, we analyzed the genomes of diverse strains that represent multiple host-specific groups (=pathotypes) of *Magnaporthe oryzae*, a fungal pathogen that infects important cereals, and related species to investigate how both types of effectors are distributed and have evolved.

Rice blast, a disease caused by *M. oryzae*, results in 10–30% yield loss every year (Talbot, 2003) and has served as a leading model for understanding the nature and mechanism of plant-pathogen interactions (Dean et al., 2012). This fungus belongs to the *Magnaporthe grisea* species complex, which also includes *M. grisea* and at least two cryptic species (Zhang et al., 2016). Individual isolates of *M. oryzae* typically have a limited host range (Choi et al., 2013b) and are divided into pathotypes based on their host range/compatibility. Since the publication of the genome sequences of *M. oryzae* strain 70-15 (Dean et al., 2005), isolates from rice and other hosts have been sequenced for comparative genomic analyses (Xue et al., 2012; Chen et al., 2013; Chiapello et al., 2015; Dong Y. et al., 2015; Gowda et al., 2015; Wu et al., 2015). Resulting data suggest that host specialization within *M. oryzae* appears to have been driven by gene gains and losses likely caused by repetitive DNA elements (Yoshida et al., 2016; Zhong et al., 2016). Other mechanisms likely underpinning host specialization include sequence change and repetitive DNA element-mediated genome rearrangement (Sánchez-Vallet et al., 2018).

The effector genes of oomycete pathogen *Phytophthora infestans* predominantly reside in gene-sparse and repeat-rich regions (Haas et al., 2009). Four *AVR* genes in *Leptosphaeria maculans*, including *AvrLm1*, *AvrLm4-7*, *AvrLm6*, and *AvrLm11*, are located at AT-rich regions (Grandaubert et al., 2014), and the *M. oryzae AVR* genes *Avr-Pita*, *Avr-Pia*, and *Avr-Pit* are located at telomere-proximal regions (Chen et al., 2007; Khang et al., 2008). Compartmentalization of effector genes in the areas of the genome that tend to change more frequently than the rest of the genome likely facilitates their rapid variation (Lo Presti et al., 2015). The ‘two-speed genome’ model was proposed to explain the bipartite genome architecture of filamentous pathogens (Dong S. et al., 2015).

The evolution of individual effector genes in *M. oryzae* has been analyzed (Dai et al., 2010; Kanzaki et al., 2012; Huang et al., 2014). Considering the large number of effector candidate (*EFC*) genes (Kim et al., 2016; Zhang et al., 2018), a systematic analysis of their distribution and genomic context across diverse isolates of *M. oryzae* is needed to understand how host adaptation has shaped the repertoire of effectors encoded by individual strains and pathotypes. We previously identified 348 *EFC* genes in *M. oryzae* strain 70-15 (Kim et al., 2016). Using this gene set, we mined their homologous CDS in 31 *M. oryzae* isolates from rice (*Oryza*), wheat (*Triticum*), foxtail (*Setaria*), goosegrass (*Eleusine*) and ryegrass (*Lolium*), three *M. grisea* strains isolated from crabgrass (*Digitaria*), and one strain each for eight species in the family Magnaporthaceae. We also mined the genomes of these isolates for the homologs of 15 known *M. oryzae AVR* genes (Table 1). The distribution pattern and genomic context of individual *EFC* and *AVR* genes were analyzed in a phylogenomic context. For comparison, we analyzed two sets of conserved 70-15 genes, including 10 reference (*REF*) genes that have been commonly used for gene expression analysis (Omar et al., 2016) and 2,245 single-copy orthologous (*SCO*) genes, and their homologs in other strains. Results from this comprehensive analysis help understand the genetic basis of host adaptation within *M. oryzae*.

**TABLE 1:** The *M. oryzae* AVR and host-specificity genes analyzed in this study.

**Gene**	**Nucleotide ID^1^**	**Protein ID^1^**	**Isolate used^2^**	***R*-gene^3^**	**References**
*Avr-Pita1*	AF207841.1	AAK00131.1	4224-7-8	*Pi-ta*	Orbach et al., 2000
*Avr-Pita2*	DQ855956.1	ABM30145.1	G-213	*Pi-ta*	Khang et al., 2008
*Avr-Pita3*	DQ855958.1	ABM30146.1	G-22	–	Khang et al., 2008
*Avr1-Co39*	AF463528.1	AAO14615.1	2539	*CO39*	Farman et al., 2002
*Avr-Pia*	AB498873.1	BAH59484.1	Ina168	*Pia*	Yoshida et al., 2009
*Avr-Pib*	KM887844.1	AKO62639.1	CHL42	*Pib*	Zhang et al., 2015
*Avr-Pii*	AB498874.1	BAH59485.1	Ina168	*Pii*	Yoshida et al., 2009
*Avr-Pik*	AB498875.1	BAH59486.1	Ina86-137	*Pikm*	Yoshida et al., 2009
*Avr-Pi9*	KM004023.1	AIS23643.1	R88-002	*Pi9*	Wu et al., 2015
*Avr-Pi54*	HF545677.2	CCN97897.1	Mo-nwi-55	*Pi54*	Ray et al., 2016
*AvrPiz-t*	EU837058.1	ACF39937.1	81278ZB15	*Piz-t*	Li et al., 2009
*Pwl1*	U36923.1	AAA80239.2	WGG-FA40	–	Kang et al., 1995
*Pwl2*	U26313.1	AAA91019.1	4392-1-6	–	Sweigard et al., 1995
*Pwl3*	U36995.1	AAA80240.1	WGG-FA40	–	Kang et al., 1995
*Pwl4*	U36996.1	AAA80241.1	WGG-FA40	–	Kang et al., 1995

*^1^Genbank accession numbers are shown. ^2^The strain used to clone each gene is noted. ^3^The resistance (*R*) gene corresponding to each *AVR*/host-specificity gene is noted.*

## Materials and Methods

### Sources of the Genomes Analyzed and the *AVR* Genes and *EFC* Genes Used to Mine Their Homologs in the Collected Genomes

We used a literature survey to collect the assembled genomes of 31 *M. oryzae* strains, including 12 MoO (representing pathotype *Oryza*), 11 MoT (*Triticum*), four MoE (*Eleusine*), three MoS (*Setaria*) and one MoL (*Lolium*) isolates, and three *M. grisea* (Mg) isolates from *Digitaria* (Supplementary Table 1). The genomes of *M. oryzae* strain 70-15, *Magnaporthe poae*, *Gaeumannomyces graminis*, and *Neurospora crassa* were downloaded from Comparative Fungal Genomics Platform (Choi et al., 2013a). The genomes of *Harpophora oryzae*, two Mg strains (DS0505 and DS9461), five MoO isolates (98-06, FJ81278, HN19311, KJ201, and MG01), two MoS isolates (SV9610 and SV9623), two MoE isolates (EI9411 and EI9604), and two MoT isolates (B71 and BdMeh16-1) were downloaded from the NCBI. The genomes of Mg isolate BR29, five MoO isolates (FR13, GY11, PH14, TH12, and TH16), MoS isolate US71, MoE isolate CD156, and MoT isolate BR32 were downloaded from GEMO-INRA (Chiapello et al., 2015). The genomes of eight MoT isolates (PY36.1, PY86.1, PY0925, PY5003, PY5010, PY5033, PY6017, and PY6045), MoL isolate PGKY, and MoE isolate BR62 were obtained from Open Wheat Blast (Islam et al., 2016). The genomes and proteomes of five species in the family Magnaporthaceae, including *Magnaporthe salvinii*, *Magnaporthiopsis incrustan*, *Magnaporthiopsis rhizophila*, *Ophioceras dolichostomum*, and *Pseudohalonectria lignicola*, were obtained from Rutgers University (Zhang et al., 2018). We used sequences of the AVR proteins downloaded from the NCBI (Table 1) and 348 small secreted proteins of MoO strain 70-15 as effector candidates (Kim et al., 2016) to identify their homologous CDSs encoded by the collected genomes.

### Genome Annotation, Repeat Annotation and Homology Search for the *AVR* and *EFC* Genes

The genomes of *H. oryzae*, two *M. grisea* and 11 *M. oryzae* isolates that did not have proteome information were annotated using Maker 2.31.8 (Cantarel et al., 2008) linked with Augustus 2.5.5 (Stanke and Morgenstern, 2005), Exonerate 2.2.0 (Slater and Birney, 2005), SNAP (Korf, 2004), CEGMA 2.5 (Parra et al., 2007), and GMHMM3 3.49 (Ter-Hovhannisyan et al., 2008) (Supplementary Table 1 and Supplementary Dataset 1)^1^. The annotated proteome data were combined with the publicly available data for the other isolates to construct the phylogenomic tree (see below). The homolog of each AVR (Table 1) or EFC protein was identified using the “protein2genome” option of Exonerate 2.2.0 (Slater and Birney, 2005). For each protein, we chose the best hit among identified CDS as its homolog. Sequence identity between each reference AVR or EFC protein and its homologs in other isolates was calculated using Sident in TrimAl 1.2 after sequence alignment using Mafft 7.273 (Capella-Gutierrez et al., 2009; Katoh and Standley, 2013). We included the coverage of aligned sequences in calculating sequence identity (Capella-Gutierrez et al., 2009). To study the genomic context of the *AVR* and *EFC* genes, we analyzed all the genomes using RepeatMasker 4.0.5 with fungal repeat library from RepBase (20160829) (Tempel, 2012).

### Clustering Analysis and Construction of the Phylogenomic and Phylogenetic Trees

The predicted proteomes of all the isolates were clustered using OrthoFinder 1.1.2 based on the default BlastP parameter and the inflation value 1.5 (Emms and Kelly, 2015). A phylogenomic tree was constructed using protein sequences of the 2,245 *SCO* genes identified via a clustering analysis with *Neurospora crassa* as an outgroup. How we performed the clustering analysis is described in Supplementary Table 2. After aligning protein sequences in each orthogroup using Mafft 7.273 (Katoh and Standley, 2013), we trimmed the aligned sequences and identified conserved regions using TrimAl 1.2 (Capella-Gutierrez et al., 2009). The sequences of individual orthogroups were concatenated to perform a phylogenetic analysis via RAxML 8.2.9 (Stamatakis, 2014). The Maximum Likelihood (ML) tree was generated using the default new rapid hill-climbing algorithm and JTTF protein model. The Neighbor-Joining (NJ) tree was constructed using MEGA 7 with 500 bootstrapping replicates (Kumar et al., 2016). Phylogenetic trees of *SCOs*, *AVRs*, and *EFCs* were constructed using Fasttree v2.1.9 (Price et al., 2010). The unrooted phylogenetic trees were rooted using the minimum variance algorithm in the MinVar-Rooting tool (Mai et al., 2017) so that we could compare individual gene trees with the phylogenomic tree. We performed this comparison using TreeKO (Marcet-Houben and Gabaldon, 2011).

### Evolutionary Diversity Analysis

The haplotype diversity (*h*) and nucleotide diversity (π) of *SCOs*, *REFs*, *AVRs*, and *EFCs* were calculated using DnaSP 6.10.01 (Rozas et al., 2017). The dN/dS ratio for each gene group was calculated using codeml in PAML 4.9e package (Yang, 1997). We used FastTree 2.1.9 (Price et al., 2010) to construct the gene trees used for calculating the average dN/dS ratio. We discarded the gene groups with the average dN/dS ratio of >10 because their high ratios were likely caused by inaccurate sequence alignment during the automatic trimming and alignment of sequences.

### Gene Expression Analysis

We analyzed transcriptome data from MoO isolate KJ201 (Jeon et al., 2019) and Bangladesh MoT isolate 12, a strain closely related to BR32 (Saunders et al., 2017). The data for KJ201 covered the mycelial stage in culture and the host infection stage at multiple time points [18, 27, 36, 45, and 72 h post-inoculation (hpi)]. The transcriptome data of Bangladesh isolate 12 were generated using field-collected wheat plants that displayed blast symptoms and those that look asymptomatic. We downloaded these data sets from the NCBI (SRA accession no. SRX5076910-SRX5076915) and Open Wheat Blast (Asymptomatic: LIB21748 and Symptomatic: LIB21752) (Saunders et al., 2017), respectively. Read mapping to the corresponding genomes was performed using HISAT2-2.0.5 (Kim et al., 2015). Because the genome of Bangladesh isolate 12 has not yet been sequenced, we used the genome of MoT isolate BR32 to map the transcriptome data. We used GFOLD v1.1.4 to conduct read counting (Feng et al., 2012).

## Results

### Distribution Pattern of the Proteins Homologous to *M. oryzae* AVRs

**FIGURE 1:**
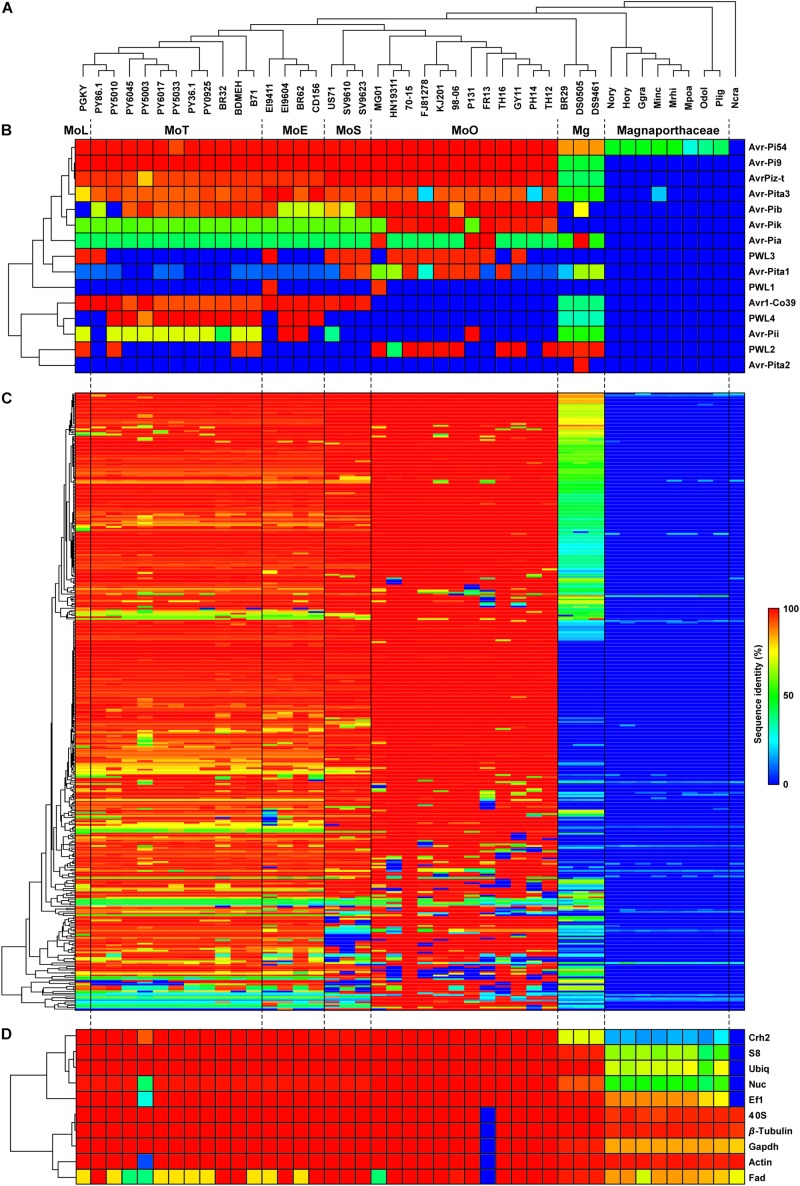
Distribution patterns of the AVR and EFC proteins encoded by *M. oryzae*, *M. grisea* and other Magnaporthaceae species. **(A)** A phylogenomic tree of the isolates/species analyzed in this study was constructed using 2,245 *SCO* gene products. The pathotype of each *M. oryzae* isolate is abbreviated according to the host of origin: *Lolium* (MoL), *Triticum* (MoT), *Eleusine* (MoE), *Setaria* (MoS), and *Oryza* (MoO). Isolates of *M. grisea* from *Digitaria* are designated as Mg. (The species in the family Magnaporthaceae are abbreviated as follows: Nory (*Nakataea oryzae*), Hory (*Harpophora oryzae*), Ggra (*Gaeumannomyces graminis*), Minc (*Magnaporthiopsis incrustans*), Mrhi (*Magnaporthiopsis rhizophila*), Mpoa (*Magnaporthiopsis poae*), Odol (*Ophioceras dolichostomum*), and Plig (*Pseudohalonectria lignicola*). *Neurospora crassa*, abbreviated as Ncra, is used as an outgroup. **(B)** A hierarchically clustered heatmap based on the degree of protein sequence identity depicts the presence/absence of the known *AVR* genes. **(C)** The heatmap for 348 EFCs, constructed using the data in Supplementary Table 4, is shown. **(D)** The heatmap for housekeeping REFs is shown.)

We mined the genes homologous to the previously characterized *AVR* (Table 1) and *EFC* (Kim et al., 2016) genes from the genomes of 31 isolates corresponding to five pathotypes of *M. oryzae*. The published annotation of the MoO strain 70-15 genome did not include the *Avr-Pia* and *Avr-Pib* genes. However, we discovered these genes via genome re-annotation (Supplementary Figure 1 and Supplementary Table 3), underscoring the importance of genome re-annotation for accurate comparative genomic studies. We analyzed how these *AVR* and *EFC* genes are distributed and structured in light of the phylogenomic relationship among the analyzed isolates. The genomes of three *M. grisea* isolates from *Digitaria* and one isolate each for eight species in the family Magnaporthaceae were also included in this analysis. A phylogenomic tree built using 2,245 *SCO* genes shows the clustering of *M. oryzae* isolates into monophyletic clades corresponding to the host of origin, and *M. grisea* isolates are distinct from the *M. oryzae* isolates (Figure 1A). The *M. oryzae* isolate tree based on a coalescent method (Gladieux et al., 2018) and the Neighbor-Joining tree (Supplementary Figure 2) also supported this pattern.

Some *AVR* genes are present in both *M. oryzae* and *M. grisea* isolates, while others are present only in a single clade/pathotype (Figure 1B). The Avr-Pi54, Avr-Pi9, AvrPiz-t, and Avr-Pita3 proteins are highly conserved in *M. oryzae*, and the sequences of their homologs in *M. grisea* have diverged from those encoded by *M. oryzae* at varying degrees. All Magnaporthaceae species carry a gene that encodes a protein exhibiting 37–51% identity to Avr-Pi54, but only *M. incrustans* appears to carry a gene encoding an Avr-Pita3-like protein. Sequences of Avr-Pib, Avr-Pik, and Avr-Pia in *M. oryzae* varied widely with the degree of identity ranging from 41 to 100% compared to the ones used to mine them. Only *M. oryzae* isolates encode Avr-Pik, but highly conserved Avr-Pia and Avr-Pib are encoded by Mg isolate DS0505. Only the MoO isolates do not encode the Avr1-Co39 protein in *M. oryzae*. Two MoE, one MoO isolate, and all MoT isolates, except PY86.1, carry a gene that encodes a protein highly similar to Avr-Pii (72% identity). For the *Avr-Pita* gene family, *Avr-Pita3* is present in all isolates, *Avr-Pita1* is mainly present in MoO and MoS isolates, and only Mg isolate DS0505 carries *Avr-Pita2*. Members of the *PWL* host-specificity gene family sporadically appeared.

### Distribution Pattern of the Proteins Homologous to the 70-15 EFCs

**FIGURE 2:**
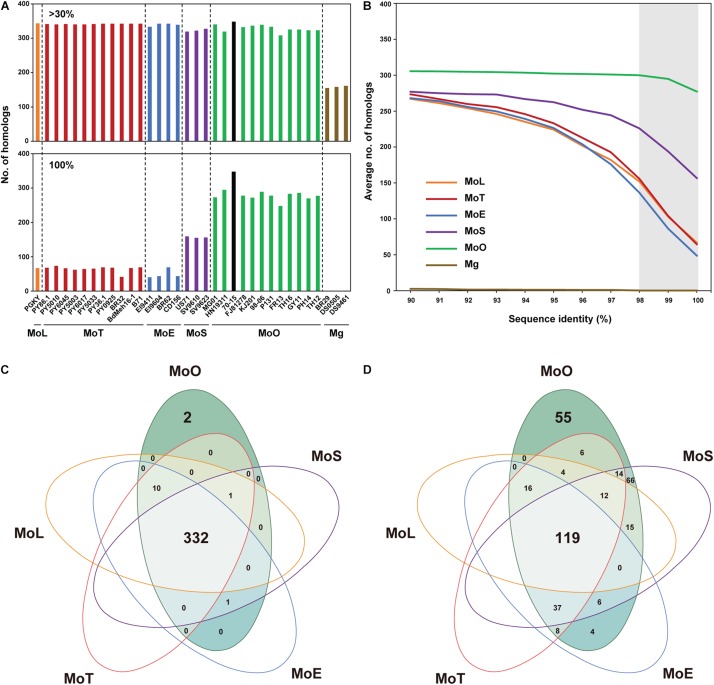
Distribution pattern of the proteins homologous to the 70-15 EFCs. **(A)** The number of EFC proteins homologous to those encoded by strain 70-15 (black bar) in each *Magnaporthe* isolate, identified via Exonerate, is shown. Data collected using two levels of sequence identity (>30% and 100%) are presented. Each pathotype is color-coded: MoL (orange), MoT (red), MoE (blue), MoS (purple), MoO (green), and Mg (brown). **(B)** The average numbers of EFCs that exhibit 90–100% sequence identity to their homologs in 70-15 are shown. The gray shading indicates those that display 98–100% sequence identity. The number of EFC homologs shared among the pathotypes at the level of **(C)** 30% and **(D)** ≥98% sequence identity is noted. The list of EFCs in each set is noted in Supplementary Table 4.

Our Exonerate-based genome re-annotation uncovered many EFC-encoding genes that could not be identified via a BlastP-based search (Figure 1C and Supplementary Figure 3). The hierarchical clustering of EFC proteins showed the pattern of distribution that is similar to that for AVRs (Figure 1B) but different from that for REFs (Figure 1D). We determined the sequence identity between the 70-15 EFCs and their homologs in each isolate (Supplementary Table 4). Both the number of EFCs in each isolate (Figure 2A) and the average number of EFCs in each pathotype (Figure 2B) were subsequently analyzed using different levels of identity as filters. At the level of >30% identity, all *M. oryzae* isolates encode homologs of most 70-15 EFCs. However, we could detect homologs in Magnaporthaceae species but found 157 homologs in Mg isolates at this level (Figure 2A). The MoL, MoT, and MoE isolates encode slightly more homologs than the MoS and MoO isolates, but the difference was small and varied among isolates within individual pathotypes. For example, 11 MoT isolates had 346–347 homologs, whereas MoO isolates FR13 and HN19314 had 320 and 329, respectively. Except for Mg, the average number of homologs in each pathotype only slightly decreased until the identity of 90% or higher was applied (Supplementary Figure 4). The numbers rapidly decreased in all pathotypes except MoO at levels higher than 90% identity (Figure 2B and Supplementary Figure 4). The average number of homologs in MoO stayed approximately 300 until up to the level of 98% identity and only slightly decreased even at the level of 100% (Supplementary Figure 4). At the level of 100% identity, the number of homologs in the MoL, MoT, and MoE isolates ranged from 40 (MoE; EI9411) to 73 (MoT; PY5010). Consistent with their phylogenomic relationship with the MoO isolates, the MoS isolates encode more homologs than the other pathotypes (Figure 2B). If we assume those with a sequence identity of >30% as homologs, 332 genes (95.4% of the total) are present in all *M. oryzae* pathotypes with only 2 (0.6%) being specific to MoO (Figure 2C). When ≥98% identity was used, 119 (34.2%) genes are present in all *M. oryzae* pathotypes (Figure 2D). Because many *EFC* gene products encoded by the Mg isolates highly diverged from those encoded by 70-15, making their comparative analyses difficult with those encoded by *M. oryzae*, we excluded them from subsequent analyses.

### Structural and Sequence Variation of the *EFC* Genes in Different *M. oryzae* Pathotypes

**FIGURE 3:**
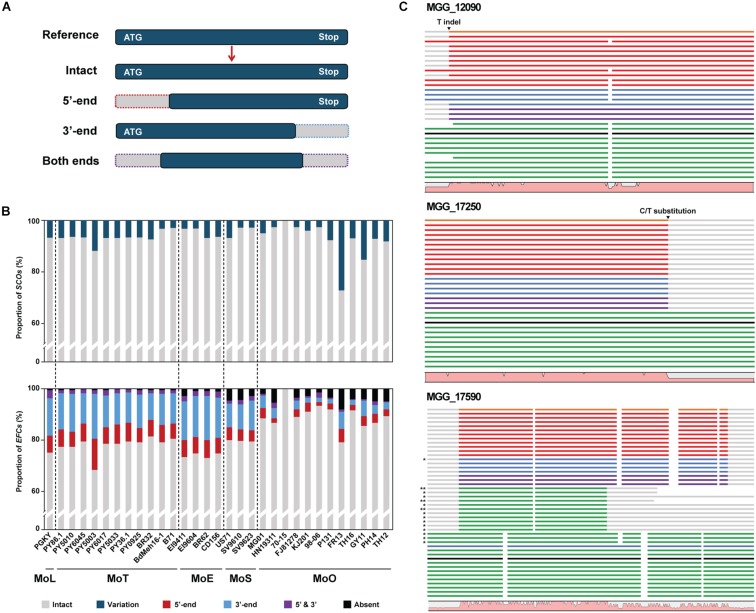
Characteristics of the structural variations in the *SCO* and *EFC* genes in *M. oryzae* isolates. **(A)** A schematic diagram depicting the types of gene structural change found using Exonerate. **(B)** The proportions of *SCO* (top) and *EFC* (bottom) genes in each isolate are shown. The proportions of the *SCOs* with structural variation (teal) and the *EFC*s detected to be truncated at 3′-end (blue), 5′-end (red) or both ends (purple) are noted. The proportion of the genes absent is noted in black. **(C)** Three examples of structural variation observed in CDS, including 5′-end variation, 3′-end variation, and variation at both ends, are shown. The isolates in the alignments follow their order in the phylogenomic tree. The reference genes encoded by MoO 70-15 are labeled black, and their homologs encoded by MoO, MoS, MoE, MoT, and MoL isolates are labeled green, purple, blue, red, and orange, respectively. For MGG_17590, asterisks are used to indicate the second (^∗^) and third (^∗∗^) best hits. The graph at the bottom of each alignment shows the degree of sequence conservation. Their full sequence alignment is shown in Supplementary Figure 5.

Certain structural changes in *AVR* genes allow pathogens to avoid recognition by host resistance gene products (Sánchez-Vallet et al., 2018). We analyzed the *EFC* genes encoded by individual *M. oryzae* isolates using Exonerate to analyze their variation within *M. oryzae* (Supplementary Table 5). Some *EFC* genes displayed variation(s) at 5′-end, 3′-end, or both ends (Figure 3A). Not surprisingly, most *SCO* genes are intact in all isolates, but we found some that appeared to exhibit structural variation compared to their homolog in 70-15 (Figure 3B). The proportion of the *EFC* genes exhibiting structural variation was larger than that of *SCOs* in all isolates except FR13. The aligned CDS of MGG_12090, MGG_17250, and MGG_17590 illustrate three types of structural variation observed (Figure 3C). In MGG_12090, an insertion or deletion of T in some isolates appears to have caused its 5′-end variation. We also found two substitutions (G/C and A/G) at both sides of the splicing sites of the second intron (Supplementary Figure 5A). We detected the indel and G/C substitution in some MoS, MoE, MoT, and MoL isolates and the A/G substitution in all pathotypes, except MoO. The 3′-end variation in MGG_17250 was caused by a C/T substitution present at one splicing site among all MoO isolates (Supplementary Figure 5B). The high degree of variation observed at the ends of MGG_17590 is due to the presence of paralogs in some isolates (Supplementary Figure 5C).

### Genomic Contexts of the *AVR* and *EFC* Genes

Based on the ‘two-speed genome model’ (Dong S. et al., 2015), we hypothesized that the *EFC* and *AVR* genes in *M. oryzae* would be located in regions that likely undergo frequent changes. We analyzed their association with repetitive elements and the distance to neighboring genes (=intergenic length) and compared observed patterns with those associated with the *SCO* genes. The *AVR* genes in 70-15 are located in repeat-rich regions and have longer 5′ and 3′ intergenic lengths compared to its *SCOs* (Supplementary Figures 1, 6). Likewise, most *EFC* genes have longer 5′- and 3′-intergenic lengths compared to the *SCOs* (Supplementary Figure 7).

**FIGURE 4:**
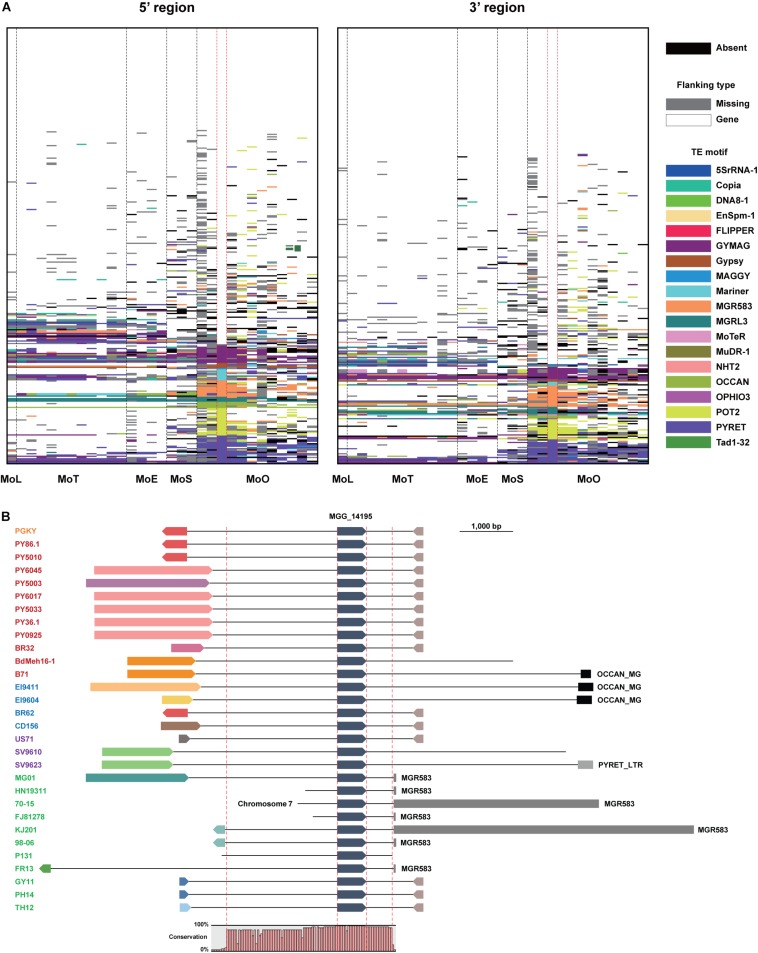
Transposable elements flanking the *EFC* genes in different *M. oryzae* isolates. **(A)** Transposable elements (TEs) present at the 5′ and 3′ regions of the *EFC* genes in different pathotypes are shown. Those present in strain 70-15 are shown between dashed red lines. The absence of each *EFC* is noted black, and the presence of a gene in its flanking regions is noted in white. If there is no element in flanking regions of EFC due to the end of a contig, it is shown in gray. Different TE motifs are denoted using multiple colors. **(B)** The flanking regions of MGG_14195 in strain 70-15 and its homologs in other *M. oryzae* isolates are shown. The isolates analyzed are color-coded: MoO (green), MoS (purple), MoE (blue), MoT (red), and MoL (orange). They were presented following the order shown in the phylogenomic (Figure 1A). The directionality of the gene and flanking genes are indicated. The conserved region in their sequences was examined using Mugsy, a genome alignment tool (Angiuoli and Salzberg, 2011). The whole contig alignment shows the region covering the gene, and its conserved flanking regions are shown between red dashed red lines (2,551 and 553 bp).

In 70-15, 40.5% of the *EFC* genes are associated with TEs, which is the highest among all isolates (Supplementary Table 6). The average proportion of the *EFCs* associated with TEs among the MoO isolates was 25.3%, but in other pathotypes, it is lower (18.9% for MoT, 16.1% for MoE, and 16.5% for MoS). Although the TEs flanking some *EFC* genes are conserved across all pathotypes/isolates analyzed, in most cases, their homologs in different isolates are associated with different TEs or lack them (Figure 4A). For example, MGG_14195 is highly conserved in all *M. oryzae* pathotypes, but its flanking regions varied a lot (Figure 4B). MGR583 is present at the 3′-region in 70-15 and some MoO isolates but is absent in other MoO isolates and different pathotypes (Figure 4B). We investigated whether the presence of TEs in flanking regions potentially influenced the degree of sequence/structural variation (Supplementary Figure 8 and Supplementary Tables 4, 5). Compared to homologs of the *EFC* genes that do not have TEs in the flanking regions in 70-15, those associated with TEs displayed higher variation (Supplementary Figure 8).

### Evolution of Some *AVR* and *EFC* Genes Looks Discordant With the Evolution of *M. oryzae*

**FIGURE 5:**
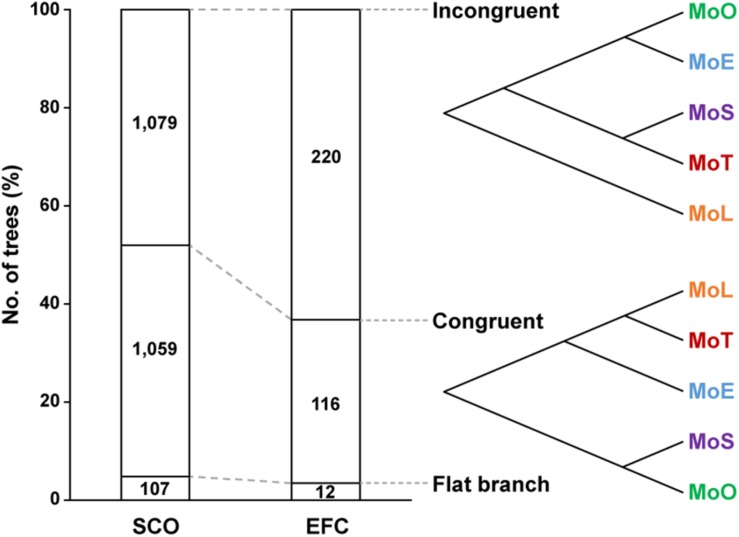
Patterns of the evolution of the *SCO* and *EFC genes.* The phylogenetic trees built using the *SCO* and *EFC* genes are grouped into those that are incongruent (*d* ≥ 0.5) with the phylogenomic tree (Figure 1A), those that are congruent (*d* < 0.5) and those caused flat branch due to perfectly conserved sequences. An example of each case is shown.

To investigate how the *EFC* genes have evolved within *M. oryzae*, we compared the 348 phylogenetic trees built using individual *EFC* genes with the phylogenomic tree shown in Figure 1A. We delineated the degree of congruence via the strict distance (*d*), which represents the similarity between two trees (Marcet-Houben and Gabaldon, 2011). Two trees are considered congruent if *d* is less than 0.5 and incongruent if *d* is between 0.5 and 1. We could not analyze approximately 4.8% of the *SCO* genes and 3.4% of the *EFC* genes because they are identical among all isolates. Some phylogenies are congruent with the evolution within *M. oryzae*, but some are not (Figure 5). The proportions of incongruent and congruent *SCO*-based phylogenies are 47.2 and 48.0%, respectively, but the proportion of incongruent phylogenies (62.2%) is much higher than that of congruent ones (33.3%) for *EFCs*. For the *AVR* genes, due to a combination of their high degrees of sequence variation and the presence of paralogs (Figure 1B), all phylogenies did not follow the phylogenomic relationship (Supplementary Figure 9). Results from the analysis of individual *AVR* genes are shown in Supplementary Figures 10–19.

### Nucleotide Polymorphisms Associated With the *AVR* and *EFC* Genes and the Selection Pressure on These Genes

**FIGURE 6:**
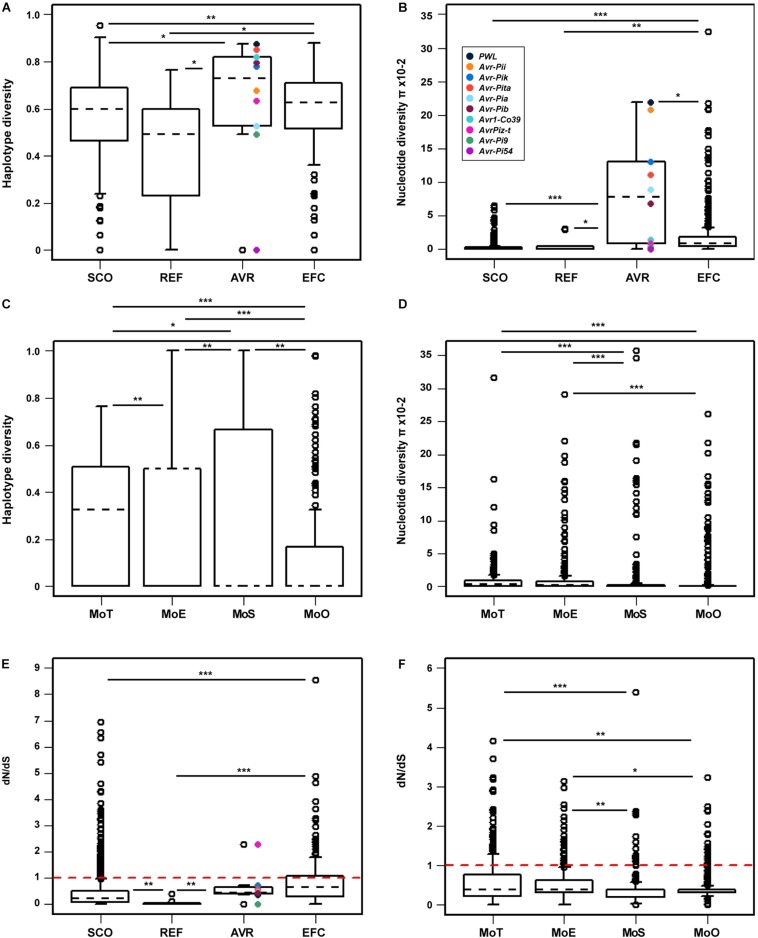
Comparison of the haplotype diversity, nucleotide diversity, and mean selection pressure among four gene sets. The **(A)** haplotype and **(B)** nucleotide diversities within each gene set are presented for comparison. The *AVR* genes are color-coded in both figures. The **(C)** haplotype and **(D)** nucleotide diversities of the *EFC* genes in four pathotypes are shown. Because only one MoL isolate was used, it was not included in this analysis. **(E)** The mean selection pressure on each of the four gene sets and **(F)** that on the *EFC* genes in four pathotypes are shown. The red dashed line in both **(E,F)** indicates neutral selection pressure (dN/dS = 1). In all figures, black dashed lines denote the median values for each gene set or pathotype. The asterisks represent significant differences in distribution according to the Wilcoxon rank sum test (^∗^*P* ≤ 0.05, ^∗∗^*P* ≤ 0.01, and ^∗∗∗^*P* ≤ 0.001).

We determined the haplotype diversity (*h*), nucleotide diversity (π), and average d*_*N*_*/d*_*S*_* ratio (ω) of the *SCOs*, *REFs*, *AVRs*, and *EFCs* to evaluate how natural selection has influenced their evolution. We also determined the *h*, π, and ω of the *EFCs* in different pathotypes to examine the likely effect of host adaptation on their evolution. The haplotype diversity of a gene of interest represents its uniqueness within a population analyzed, and the nucleotide diversity represents average nucleotide differences among sampled DNA sequences (Nei, 1987). The Wilcoxon rank sum statistical test indicated that the haplotype and nucleotide diversities of *AVRs* and *EFCs* are higher than those of *SCOs* and *REFs* (Figures 6A,B). Among the *AVRs*, *PWL* exhibited the highest haplotype and nucleotide diversities, and *Avr-Pi54* displayed no variation. The nucleotide diversities of most *AVRs*, including *PWL*, *Avr-Pii*, *Avr-Pik*, *Avr-Pita*, *Avr-Pia*, and *Avr-Pib*, are similar to those displayed by the outliers of *EFC*s (Figure 6B). The haplotype diversity distribution of *EFCs* among four pathotypes varied significantly (Figure 6C), but the nucleotide diversity distribution was different only between MoT/MoE and MoS/MoO (Figure 6D).

The average ω ratio estimates selection pressure on a set of homologous genes (Yang and Bielawski, 2000). The ω ratio of one indicates neutral selection. We hypothesized that the *EFCs* would have ω values higher than one due to the selection pressure exerted by hosts. The average ω ratios for *EFCs* and *AVRs* are significantly higher than those for *SCOs* and *REFs*, but no *EFCs* display the value higher than one (Figure 6E). Among the *AVRs*, only *AvrPiz-t* displayed the ω ratio higher than one (positive selection). The average ω ratio for most *EFCs* in different pathotypes was also less than one, but some have a ω ratio higher than one (Figure 6F). The selection pressure on *EFCs* did not appear constant across the pathotypes (Supplementary Figure 20). The average ω ratios for *EFCs* in MoT and MoE are significantly higher than those in MoS and MoO isolates.

### Expression Patterns of *EFC* Genes

**FIGURE 7:**
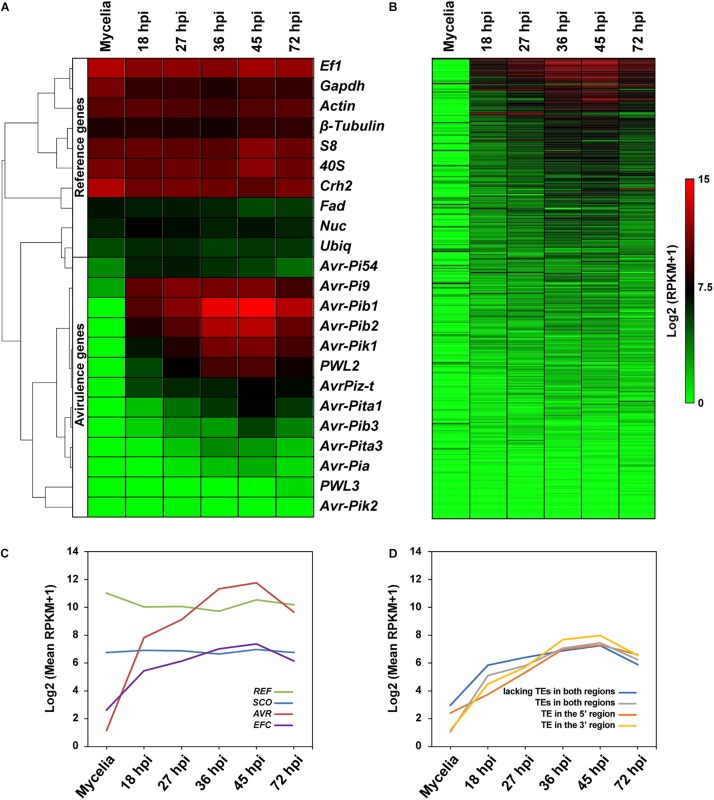
Comparative analysis of the expression patterns of the *REF*, *AVR*, *EFC*, and *SCO* genes in MoO isolate KJ201. Expression patterns of **(A)** *REFs* and *AVRs* and **(B)** *EFCs* in KJ201 in mycelia and during rice infection are shown. Data from the samples collected at the pre-penetration (18 hpi), biotrophic growth (27–36 hpi), and necrotrophic (45–72 hpi) stages are shown. **(C)** Averaged expression patterns of the four gene sets are shown. **(D)** Averaged expression patterns of the following types of *EFCs* are shown: those with TEs in both the 5′ and 3′ regions, those with TEs in the 5′ region only, and those with TEs in the 3′ region only, and those lacking TEs in both regions.

Earlier studies showed that most effector genes are exclusively expressed during infection (Stergiopoulos and De Wit, 2009). We determined if the *EFC* genes are preferentially expressed during host infection using publicly available data (Figure 7A and Supplementary Figure 21). We also analyzed expression patterns of *SCOs*, *REFs*, and *AVRs*. As expected, the expression of *REFs* in MoO isolate KJ201 did not change much during mycelial growth and infection, whereas the *AVRs* showed elevated expression during the biotrophic and early necrotrophic stages of infection (27–45 hpi) (Figure 7A). In wheat plants infected with Bangladesh isolate 12, the *REFs* were similarly expressed in both symptomatic and asymptomatic plant tissues, whereas the *AVRs* were expressed only in symptomatic tissues (Supplementary Figure 21A). The expression of most *EFCs* in KJ201 appeared elevated during the biotrophic stage compared to the mycelial growth stage (Figure 7B). Overall, the putatively infection-related gene sets (*AVRs* and *EFCs*) in both isolates showed elevated levels of expression during infection (Figure 7C and Supplementary Figure 21B). In contrast, the expression of the *SCO* and *REF* genes in KJ201 did not change significantly. We also examined whether the presence of TEs in flanking regions affects the expression of *EFCs* in KJ201 (Figure 7D). The presence or absence of TEs does not appear to have a substantial effect on their expression.

## Discussion

The host range of a pathogen is determined by its effector repertoire, a characteristic that has been used to differentiate strains/isolates within species (Schulze-Lefert and Panstruga, 2011). Most effectors in plant pathogens exhibit species-specific presence/absence or sequence features likely due to the co-evolution between pathogens and hosts (Sonah et al., 2016). Many fungal/oomycete *EFC* genes have been identified based on one or more of the following characteristics of the gene or gene product: (a) likely secretion based on the presence of a signal peptide but no transmembrane domain or GPI-anchor sites; (b) small size (usually fewer than 300 amino acids); (c) presence only in specific species or isolates; (d) increased expression during infection; (e) rich in cysteine residues; and (f) presence of a conserved motif, particularly for oomycete EFCs (Godfrey et al., 2010; Zuccaro et al., 2011; Cheng et al., 2014). We previously identified and analyzed EFCs encoded by diverse groups of fungal pathogens based on the hypothesis that small secreted proteins function as EFCs (Kim et al., 2016). In this study, we analyzed the distribution, structure, genomic context, and expression pattern of the *EFC* genes, as well as known *AVR* genes, in the isolates that represent five pathotypes of *M. oryzae* and nine related species. Although resulting data are not sufficient to validate their role as effectors, they should facilitate subsequent studies needed to validate their function and will also support research on the genetic basis of the evolution and host specialization in *M. oryzae*. All the results from this study are readily available to guide such studies, and the approaches/methods we used can be applied to mine EFCs from newly sequenced *M. oryzae* genomes.

Comparative genomic analyses of the *M. oryzae* genes that are likely involved in host specificity have been performed using publicly available genome sequence data (Yoshida et al., 2016; Zhong et al., 2016). Because multiple methods/approaches have been used to annotate the genomes of *M. oryzae* isolates, the quality of genome annotation varied, likely affecting the quality of downstream analyses based on such genome data. To ensure accurate comparative analyses of the *AVR* and *EFC* genes in *M. oryzae* and related species, we first re-annotated the genomes of diverse isolates that represent five pathotypes of *M. oryzae* and nine related species (Supplementary Table 1) via Exonerate to identify their homologs. Through this reannotation, we discovered the *Avr-Pia* and *Avr-Pib* genes in the 70-15 genome (Supplementary Figure 1 and Supplementary Table 3) and many missing homologs of the 70-15 *EFC* genes (Kim et al., 2016) in other genomes. These results underscore the importance of re-annotating chosen genomes for robust comparative genomic studies.

Using the re-annotated data, we analyzed the distribution, sequence variation, and genomic context of the *AVR* and *EFC* genes at multiple phylogenomic levels to uncover notable patterns in light of the evolution of *M. oryzae* and related species. In parallel, we also analyzed a large number of *SCO* genes that are expected to be conserved among these species to compare results with those derived from the *AVR* and *EFC* genes. Despite a few shortcomings noted below, results from this study provide a global snapshot of how the *AVR* and *EFC* genes and their products have evolved in *M. oryzae* and related species. The comprehensive catalog of the *EFC* and *AVR* genes will serve as a foundation for future studies of these genes.

In *M. oryzae*, the host range of individual isolates is typically narrow. Isolates originated from a specific host infect the host of origin and closely related species (Kato et al., 2000; Oh et al., 2002; Murakami et al., 2003) and are grouped into pathotypes. We show that many *EFC* genes are shared among the isolates in multiple pathotypes with high levels of sequence identity. Many *M. oryzae EFC* genes appear species-specific (Figures 1, 2 and Supplementary Figure 3). However, because the Mg isolates carry genes that encode products with low sequence identity to *M. oryzae* EFC proteins (Supplementary Figure 4), it is possible that such Mg *EFC* genes correspond to the orthologs of 70-15 *EFCs* that have rapidly diverged.

Host range variation within *M. oryzae* likely involves changes in effector genes, including those that encode AVR proteins. Recently, a population genetic study using *M. oryzae* isolates from rice showed that individual isolates could infect only specific lines of rice due to the variation in their effector repertoire (Liao et al., 2016). In addition to varying degrees of sequence variation in individual *EFC* genes, multiple types of structural variation were also detected in *M. oryzae* (Figure 3). Such changes likely cause pseudogenization, leading to the production of non-functional EFC proteins. Although most of these mutations seem to be phylogenetically concordant with the evolution of *M. oryzae*, a few seem to have sporadically emerged in multiple pathotypes. We cannot rule out the possibility that errors during the genome assembly and annotation of some isolates caused some of the variations observed. Besides, some genes appear to be gene family members, and we may have retrieved paralogous members during genome mining.

Previous studies reported that variations of host compatibility genes were often associated with the presence or activity of TEs (Kang et al., 2001; Khang et al., 2008), and the selection pressure from hosts likely favors such changes (Yoshida et al., 2016; Zhong et al., 2016). Our analysis shows that sequence variation among the *EFC* genes occurred more frequently than the *SCO* genes (Supplementary Figure 8), supporting the role of the former group in host interaction. To determine whether the TEs surrounding *EFC* genes contribute to their sequence/structural variation, we compared the degree of sequence variation and the proportion of genes with structural variation among the *EFC* genes after dividing them based on the presence of flanking TEs (Supplementary Figure 8). Although it remains to be determined whether associated TEs caused sequence or structural variation in individual genes, higher degrees of variation were observed among those that are associated with TEs in 70-15. We tested whether the TEs in flanking regions of homologous *EFC* genes are conserved within a species. The nature of flanking TEs is quite variable (Figure 4), suggesting that TEs or surrounding genome sequences undergo frequent changes.

Although the ω ratio of most *EFC* genes within each pathotype was less than one, suggesting purifying selection, 21.6% of the *EFC* genes in MoO and 27.1% of MoT *EFC* genes exhibited the ω ratio higher than one, suggesting that they have been positively selected (Figure 6). These genes under positive selection from host likely involved in host adaptation and specialization. However, the ω ratio of most *EFC* genes is less than one, and these genes may encode effectors that function in different hosts of *M. oryzae*. The ω ratio of *EFC* genes was not constant in each pathotype (Supplementary Figure 20) probably because the type of selection on effectors from rice is likely different from that from other hosts.

Expression of effector genes is generally induced during host-pathogen interactions (Lo Presti et al., 2015; Sánchez-Vallet et al., 2018). We used gene expression data derived from mycelial culture and infected rice and wheat plants to determine whether the *M. oryzae EFC* genes are differentially expressed during infection. Although the expression level of *EFC* genes increased during infection in both plants, the degree of induction in KJ201 appeared much higher than that in MoT isolate (Figure 7). However, additional experiments with more biological repeats and under identical environmental conditions are needed to validate this observation because the transcriptome data for KJ201 were obtained via rice sheath assay to enrich the quantity of fungal RNAs (Jeon et al., 2019), while the data from wheat plants were obtained samples collected in the field (Saunders et al., 2017). We also determined whether the presence of TEs in flanking regions of the *EFC* genes affect their expression, as some TEs have been suggested to affect the expression of *EFC* genes in other pathogens (Whisson et al., 2012). We found no significant changes potentially associated with the presence of TEs in flanking regions in KJ201 (Figure 7D). Because we did not consider the distance between each *EFC* gene and neighboring TEs, it is premature to discount the effect of TEs on the expression of *EFC* genes.

## Data Availability Statement

The datasets generated for this study can be found in the NCBI SRX5076910, SRX5076911, SRX5076912, SRX5076913, SRX5076914, and SRX5076915.

## Author Contributions

K-TK and Y-HL designed the study. K-TK, SK, and Y-HL wrote the manuscript. K-TK, JK, HS, GC, HK, JJ, and KC performed the data mining and analyses.

## Conflict of Interest

The authors declare that the research was conducted in the absence of any commercial or financial relationships that could be construed as a potential conflict of interest.

## Footnotes

1 https://figshare.com/s/5a242921965dcf5006f2

## Abbreviations

AVRs: avirulence genes
CDS: coding sequence
EFCs: effector candidate genes
Mg: M. grisea
MoE: M. oryzae pathotype Elusine
MoL: M. oryzae pathotype Lolium
MoO: M. oryzae pathotype Oryza
MoS: M. oryzae pathotype Setaria
MoT: M. oryzae pathotype Triticum
REFs: reference genes
SCOs: single-copy orthologous genes
TEs: transposable elements
