# Supplementary material for: Evolution of the Genes Encoding Effector Candidates Within Multiple Pathotypes of Magnaporthe oryzae
Source: Front Microbiol. 2019 Nov 6;10:2575. doi: 10.3389/fmicb.2019.02575 (PMC6851232; doi:10.3389/fmicb.2019.02575)
Supplement: Supplementary file 1 [file Data_Sheet_1.pdf]

# *Supplementary Material*

## Supplementary Table 1. The genomes analyzed in this study

See the corresponding excel file.

## Supplementary Table 2. Results from a proteome clustering analysis

| Orthogroups | No. of clusters | No. of proteins | Note |
|---|---|---|---|
| Total | 19,273 | 517,862 | |
| *M. grisea* species complex | 15,848 | 502,298 | No. of clusters including either Mo/Mg |
| Pan-*M. oryzae* | 15,083 | 499,617 | No. of clusters including Mo |
| Pan-*M. grisea* | 10,957 | 440,605 | No. of clusters including Mg |
| Magnaporthaceae-specific | 12,040 | 203,022 | No. of clusters without *N. crassa* |
| *Magnaporthe*-specific | 6,483 | 89,858 | No. of clusters with only Mo/Mg |
| *M. oryzae*-specific | 4,358 | 47,949 | No. of clusters with only Mo |
| MoO-specific | 485 | 1,481 | No. of clusters with only MoO |
| MoT-specific | 95 | 329 | No. of clusters with only MoT |
| MoE-specific | 20 | 40 | No. of clusters with only MoE |
| MoS-specific | 13 | 26 | No. of clusters with only MoS |
| *M. grisea*-specific | 671 | 1,920 | No. of clusters with only Mg |
| Singletons | - | 18,716 | |
| Single copy orthologs | 2,245 | 94,290 | No. of clusters with only one gene from each isolate/species |

Mo (*M. oryzae*); Mg (*M. grisea*); MoO (Mo isolates from rice); MoT (Mo isolates from *Triticum*); MoE (Mo isolates from *Eleusine*); and MoS (Mo isolates from *Setaria*)

### Proteome clustering analysis

We performed a proteome clustering analysis to find single copy orthogroups **(Supplementary Table 2)**. The proteome of *Neurospora crassa* was used as an outgroup. In total, 19,273 orthogroups and 18,716 singletons were identified. We used 2,245 single copy orthogroups to construct phylogenomic species trees.

## Supplementary Table 3. Re-annotated *AVR* genes

See the corresponding excel file.

**Supplementary Table 4. Sequence identity of the *EFC* gene products encoded by the isolates mined in this study compared to their homologs encoded by strain 70-15**

See the corresponding excel file.

**Supplementary Table 5. Structural variations observed in the *EFC* and single copy ortholog (*SCO*) genes analyzed in this study**

See the corresponding excel file.

**Supplementary Table 6. Genomic contexts of the *EFC* genes associated with transposable elements**

See the corresponding excel file.

**Supplementary Dataset 1. Genome annotation data performed in this study**

See the corresponding compressed files (https://figshare.com/s/5a242921965dcf5006f2). It includes GFF annotation and transcript files.

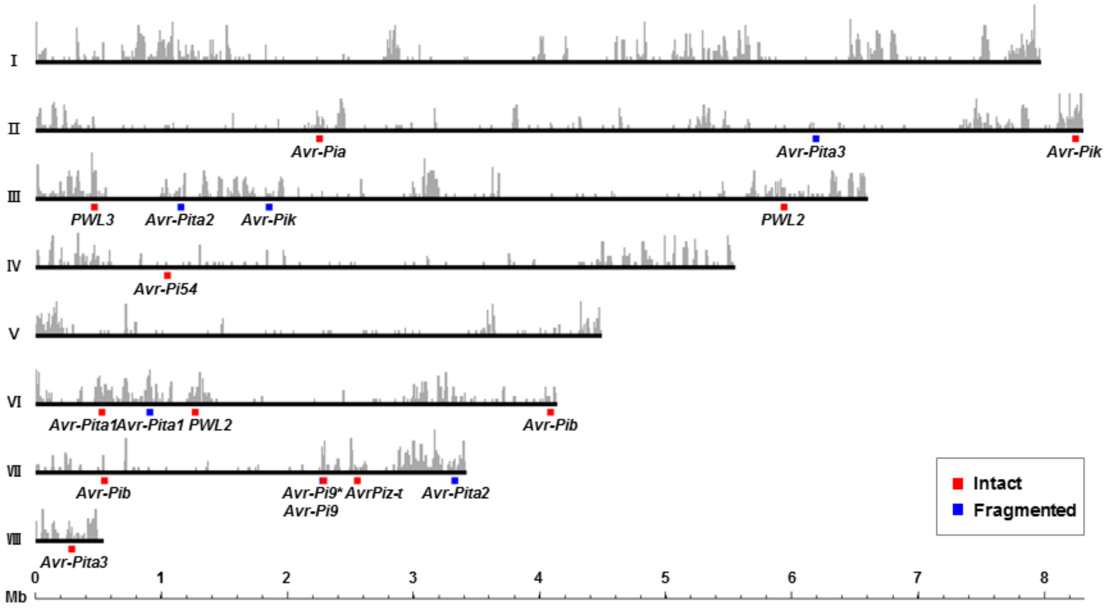


**Supplementary Figure 1. Chromosomal locations of the re-annotated *AVR* genes in strain 70-15 and the density of repetitive DNA elements**

The intact (red) and fragmented (blue) *AVR* genes and the density of repetitive elements (grey bars) in 10 kb windows are noted on the eight chromosomes of 70-15.

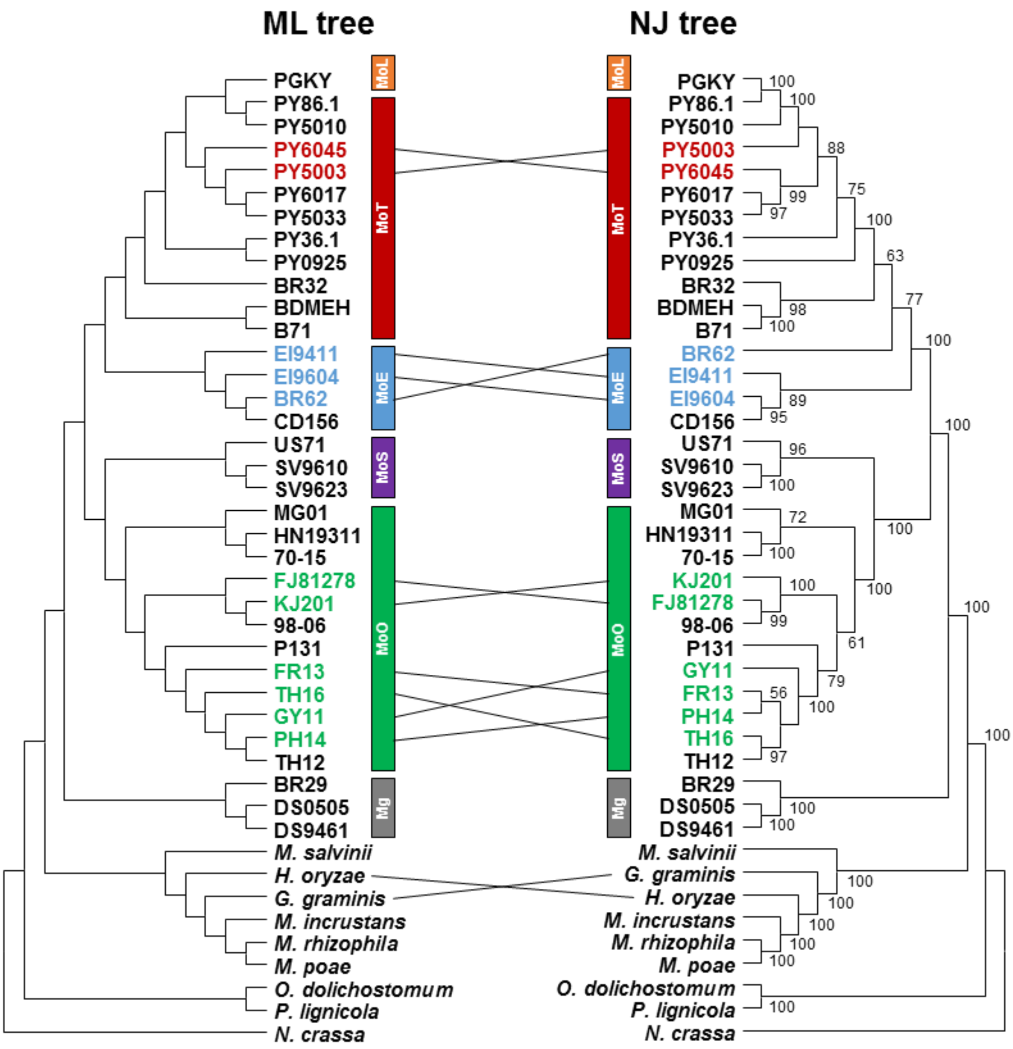


**Supplementary Figure 2. Phylogenomic trees of the *M. oryzae* isolates and those representing outgroup species analyzed in this study**

Concordance of their phylogenetic relationship in the Maximum-likelihood (ML) and Neighbor-joining (NJ) trees is shown. All *M. oryzae* isolates from each host belong to one clade. The isolates are not concordant between the trees are noted by solid lines. Isolates from different plants are color-coded. For the NJ tree, the bootstrap values over 50 % are shown.

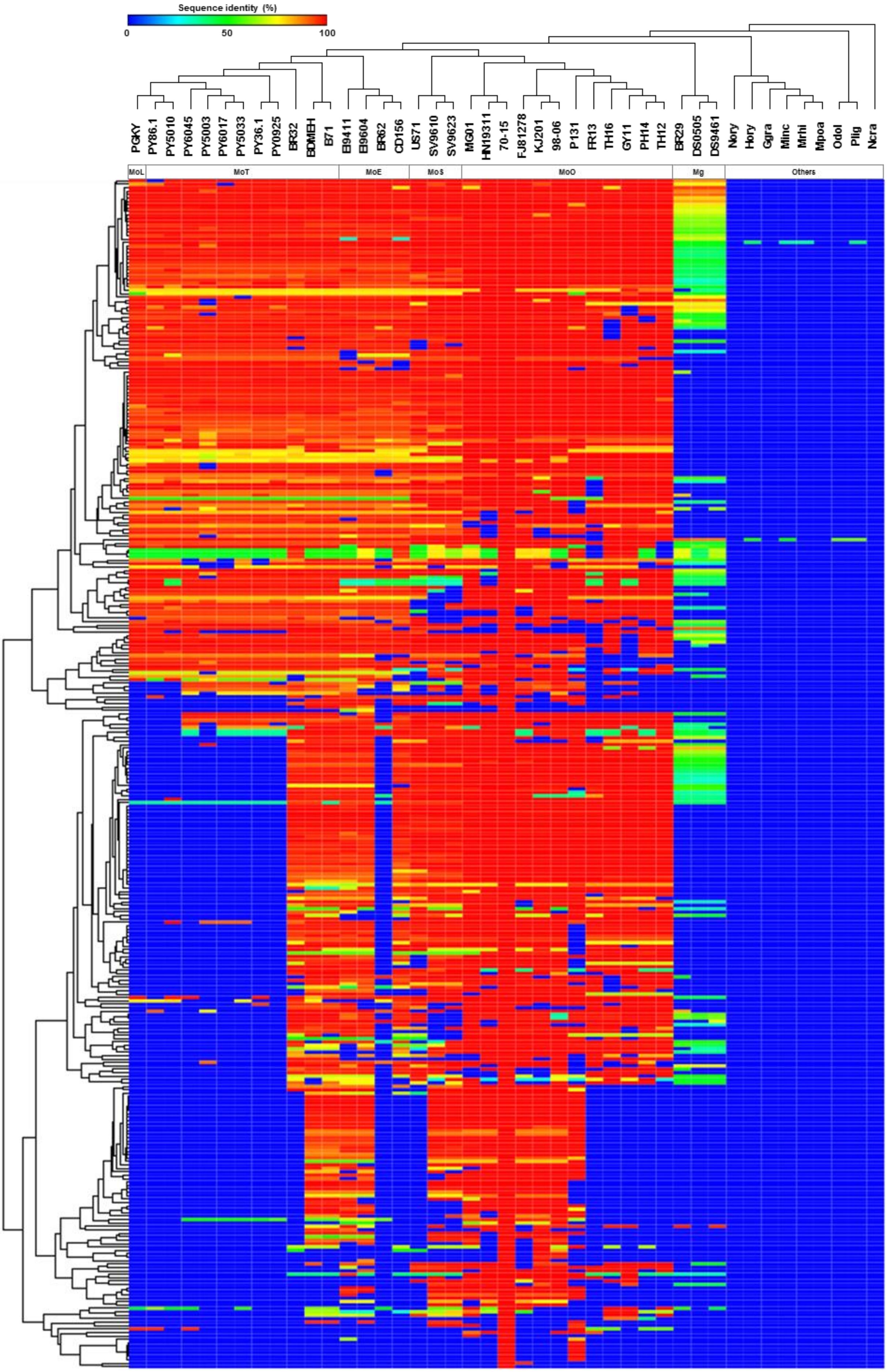
Sequence identity (%)
0
50
100
PGKY
PY86.1
PY5010
PY6045
PY5003
PY6017
PY5033
PY36.1
PY0925
BR32
BDMEH
B71
EI9411
EI9604
BR62
CD156
US71
SV9610
SV9623
MG01
HN19311
70-15
FJ81278
KJ201
98-06
P131
FR13
TH16
GY11
PH14
TH12
BR29
DS0505
DS9461
Nory
Hory
Ggra
Minc
Mrhi
Mpoa
Odol
Plig
Ncra
MoL
MoT
MoE
MoS
MoO
Mg
Others

**Supplementary Figure 3. Comparative analysis of the presence/absence and the degree of sequence identity of the EFC proteins identified via BlastP**

The heatmap depicts the degree of sequence identity of the 348 EFC proteins encoded by the *M. oryzae* and *M. grisea* isolates and those representing other Magnaporthaceae species analyzed in this study compared to their homologs encoded by strain 70-15.

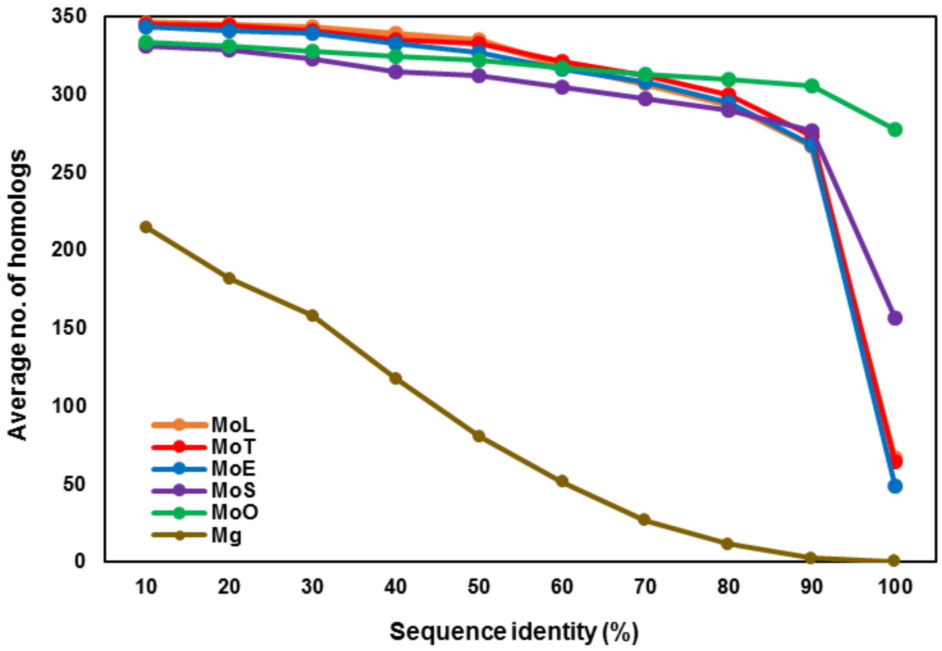


**Supplementary Figure 4. Average numbers of the EFCs in five pathotypes of *M. oryzae* and *M. grisea* calculated at different levels of sequence identity**

Results from each *M. oryzae* pathotype are color-coded: orange (MoL), red (MoT), blue (MoE), purple (MoS), and green (MoO). Brown represents data derived from *M. grisea* (Mg).

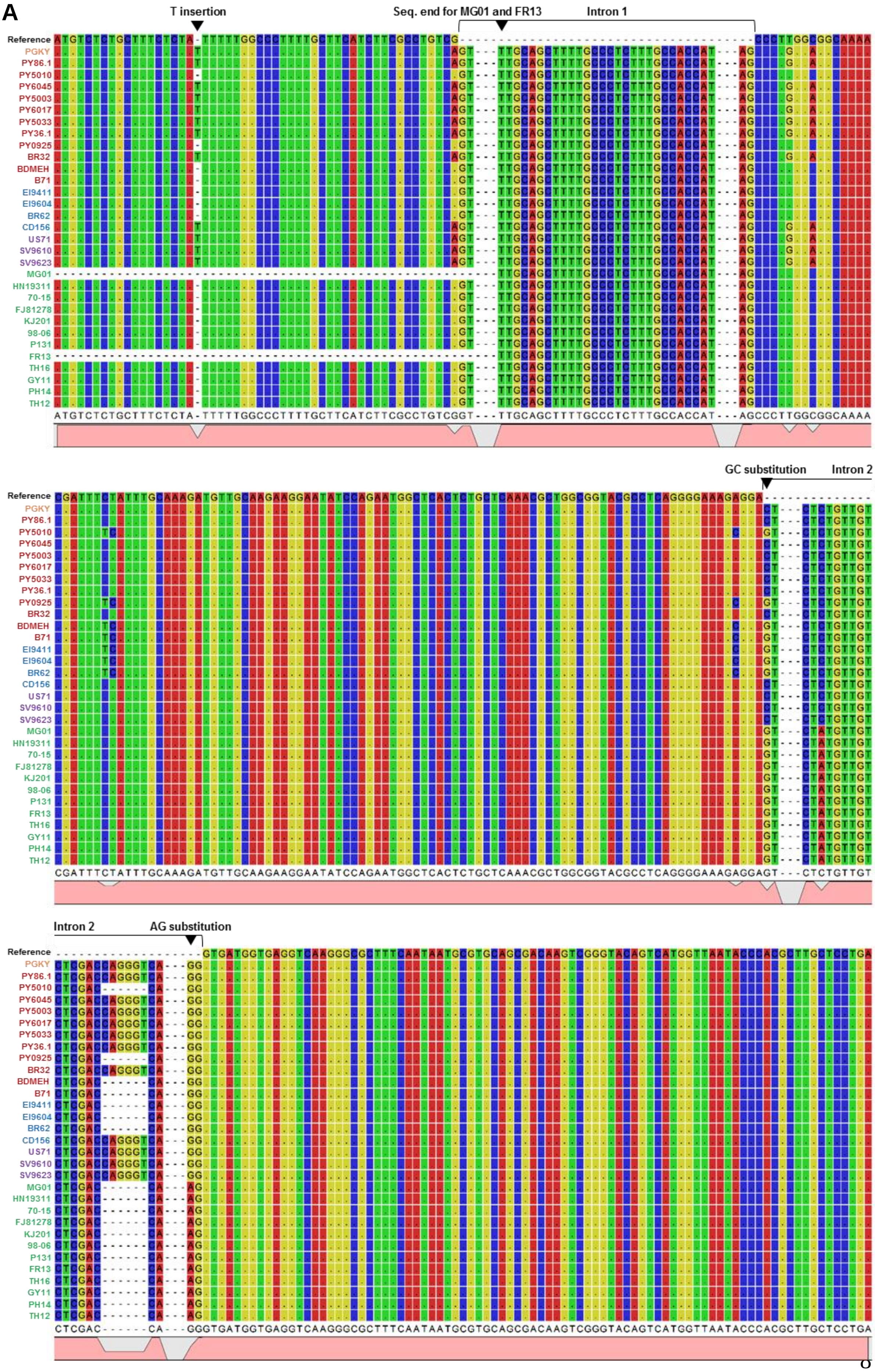
A
T insertion
Seq. end for MG01 and FR13
Intron 1
GC substitution
Intron 2
Intron 2
AG substitution
Reference
PGKY
PY86.1
PY5010
PY6045
PY5003
PY6017
PY5033
PY36.1
PY0925
BR32
BDMEH
B71
EI9411
EI9604
BR62
CD156
US71
SV9610
SV9623
MG01
HN19311
70-15
FJ81278
KJ201
98-06
P131
FR13
TH16
GY11
PH14
TH12
ATGTCTCTGCTTTCTCTA-TTTTTGGCCCTTTTGCTTCATCTTCGCCTGTCGGT---TTGCAGCTTTTGCCCTCTTTGCCACCAT---AGCCCTTGGCGGCAAAA
CGATTTCTATTTGCAAAGATGTTGCAAGAAGGAATATCCAGAATGGCTCACTCTGCTCAAACGCTGGCGGTACGCCTCAGGGGAAAGAGGAGT---CTCTGTTGT
CTCGAC------CA---GGGTGATGGTGAGGTCAAGGGCGCTTTCAATAATGCGTGCAGCGACAAGTCGGGTACAGTCATGGTTAATACCCACGCTTGCTCCTGA

**B**

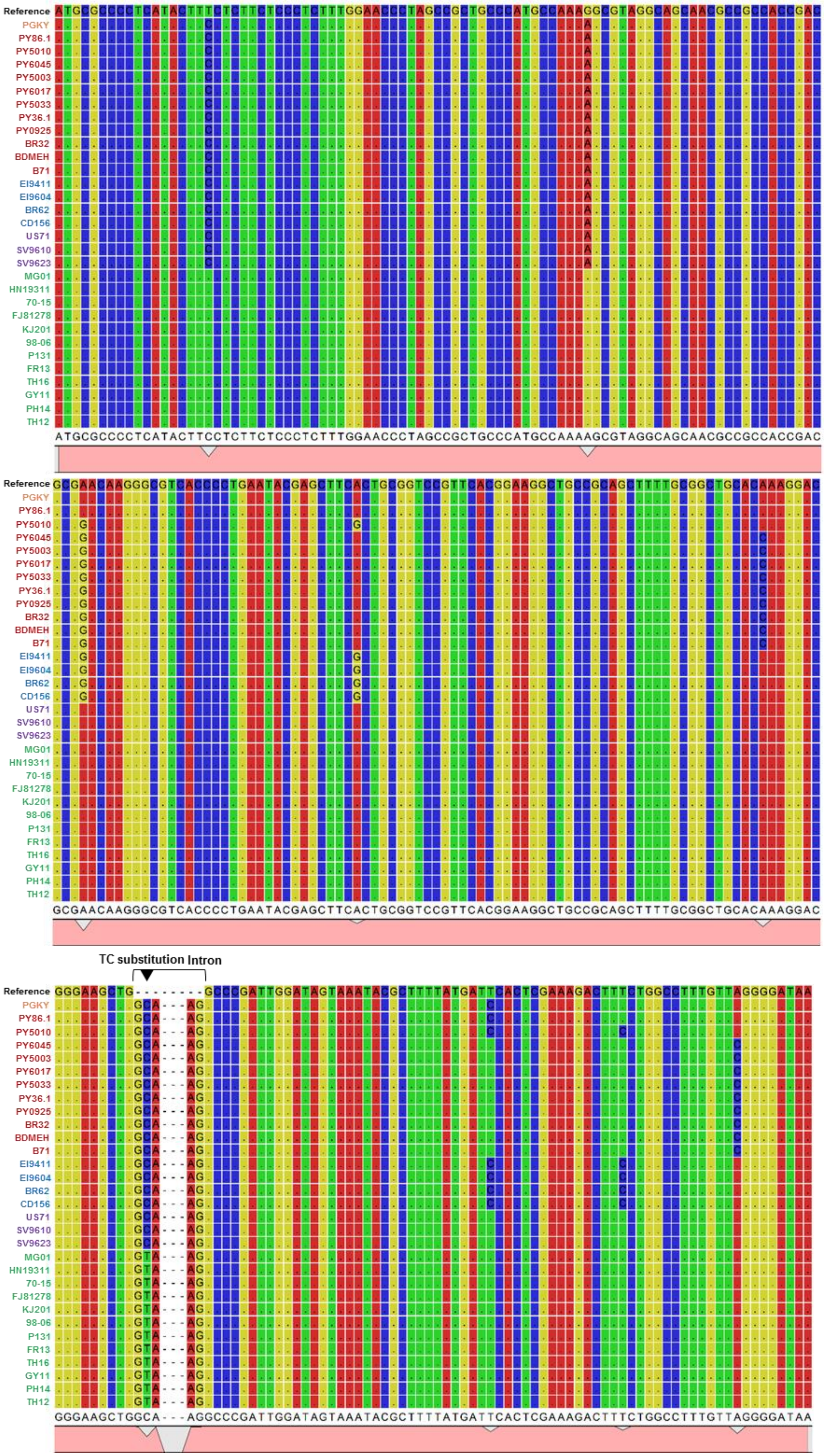
Reference
PGKY
PY86.1
PY5010
PY6045
PY5003
PY6017
PY5033
PY36.1
PY0925
BR32
BDMEH
B71
EI9411
EI9604
BR62
CD156
US71
SV9610
SV9623
MG01
HN19311
70-15
FJ81278
KJ201
98-06
P131
FR13
TH16
GY11
PH14
TH12
ATGCGCCCCTCATACTTCCTCTTCTCCCTCTTTGGAACCCTAGCCGCTGCCCATGCCAAAAGCGTAGGCAGCAACGCCGCCACCGAC
GCGAACAAGGGCGTCACCCCTGAATACGAGCTTCACTGCGGTCCGTTCACGGAAGGCTGCCGCAGCTTTTGCGGCTGCACAAAGGAC
TC substitution Intron
GGGAAGCTGGCA---AGGCCCGATTGGATAGTAAATACGCTTTTATGATTCACTCGAAAGACTTTCTGGCCTTTGTTAGGGGATAA

C

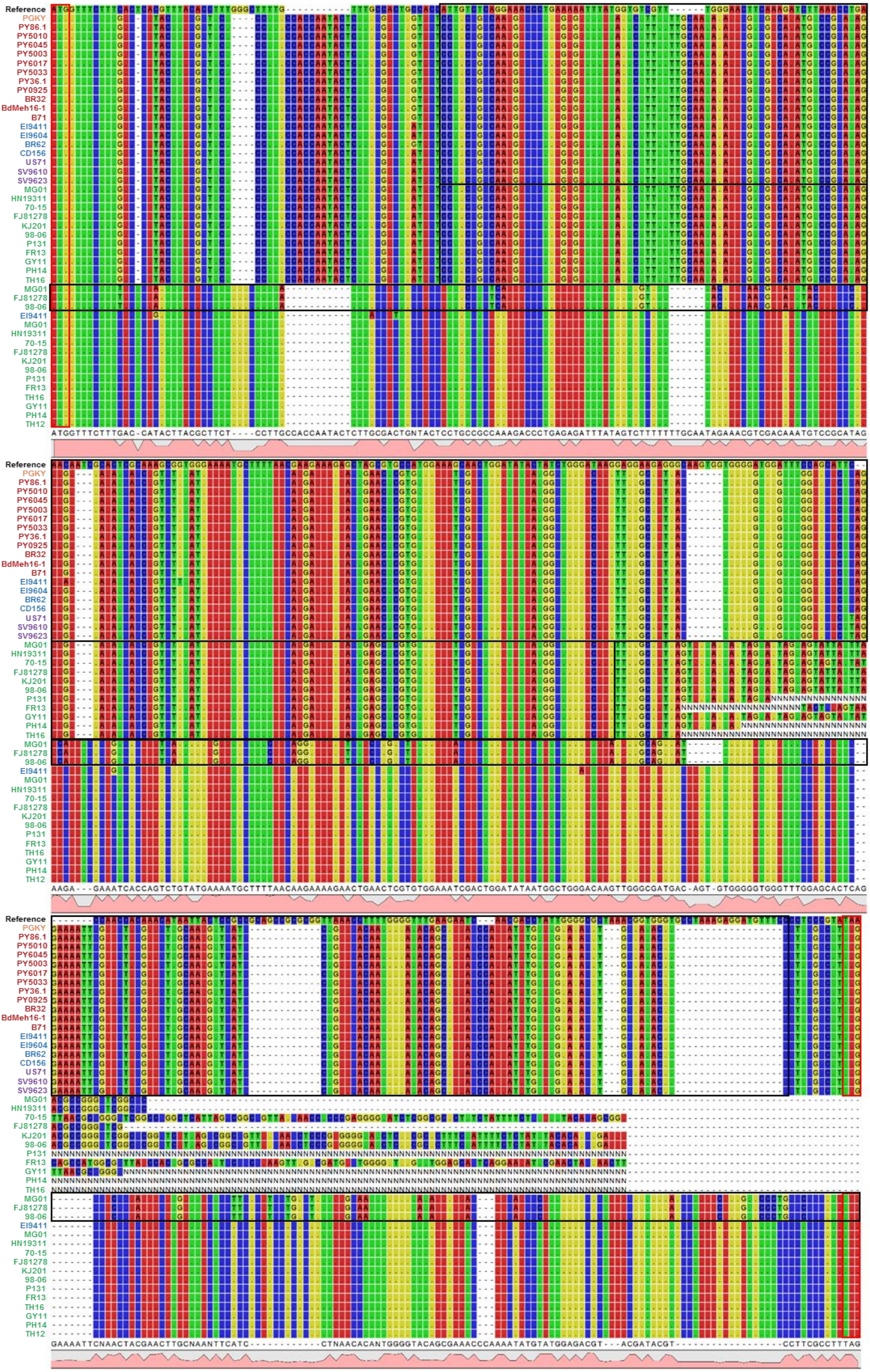
Reference
PGKY
PY86.1
PY5010
PY6045
PY5003
PY6017
PY5033
PY36.1
PY0925
BR32
BdMeh16-1
B71
EI9411
EI9604
BR62
CD156
US71
SV9610
SV9623
MG01
HN19311
70-15
FJ81278
KJ201
98-06
P131
FR13
GY11
PH14
TH16
TH12

**Supplementary Figure 5. Aligned sequences of three *EFC* genes encoded by diverse *M. oryzae* isolates.**

The aligned sequences of **(A)** MGG_12090, **(B)** MGG_17250 and **(C)** MGG_17590 genes are shown. The isolates included in this analysis are color-coded: MoO (green), MoS (purple), MoE (blue), MoT (red) and MoL (orange). Sequences of 70-15 are shown at the top, and the order of isolates presented follows the phylogeny in Figure 1A. Sequence changes that caused structural variation in each gene are noted. The introns in MGG_12090 ang MGG_17250 are noted, but their full sequences are not shown. The consensus sequences and their degree of conservation are shown at the bottom of each alignment. For **(A)**, the end of sequences from MoO isolates MG01 and FR13 corresponds to the end of a contig that contains the gene. For **(C)**, the start and stop codons are marked using red box, and highly conserved sequences are also boxed (black).

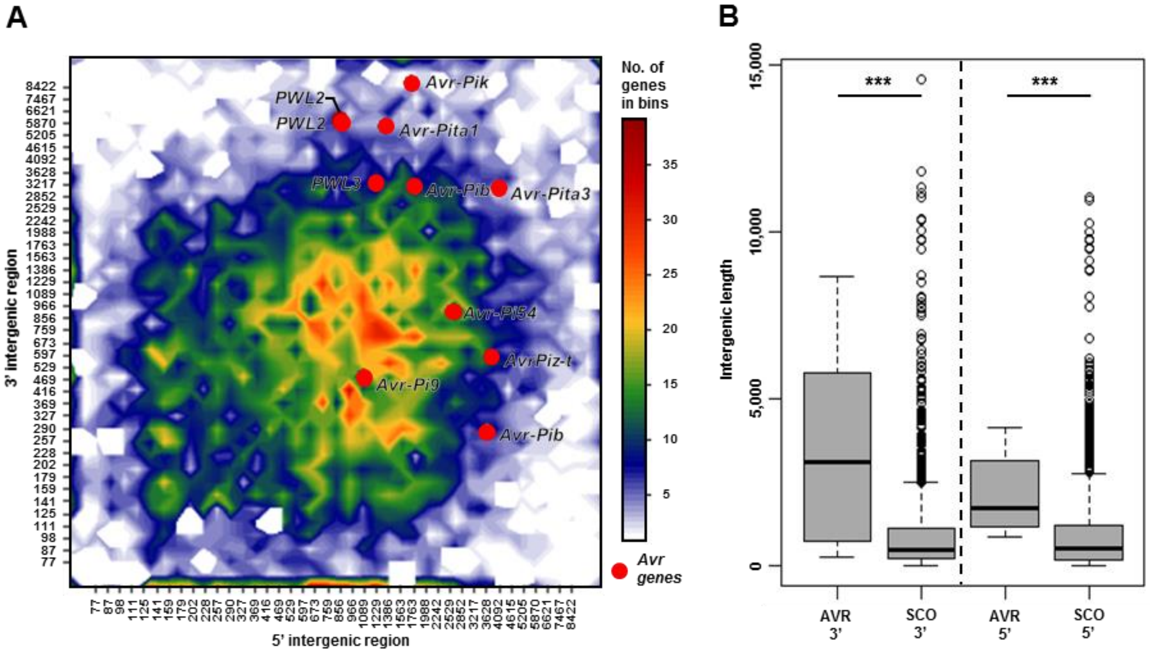


**Supplementary Figure 6. Comparison of the intergenic lengths between the known *AVR* and *SCO* genes in strain 70-15**

**(A)** The intergenic lengths of the *AVR* genes in 70-15 within a whole genome. The number of genes is color-coded via heatmap density. The position of each *AVR* gene is noted using a red dot. **(B)** Distribution patterns of the 3'- and 5'-intergenic lengths of the *AVR* genes and the single copy orthologous genes are shown. The significant difference was tested by Wilcoxon rank sum test (*** $P \leq 0.001$).

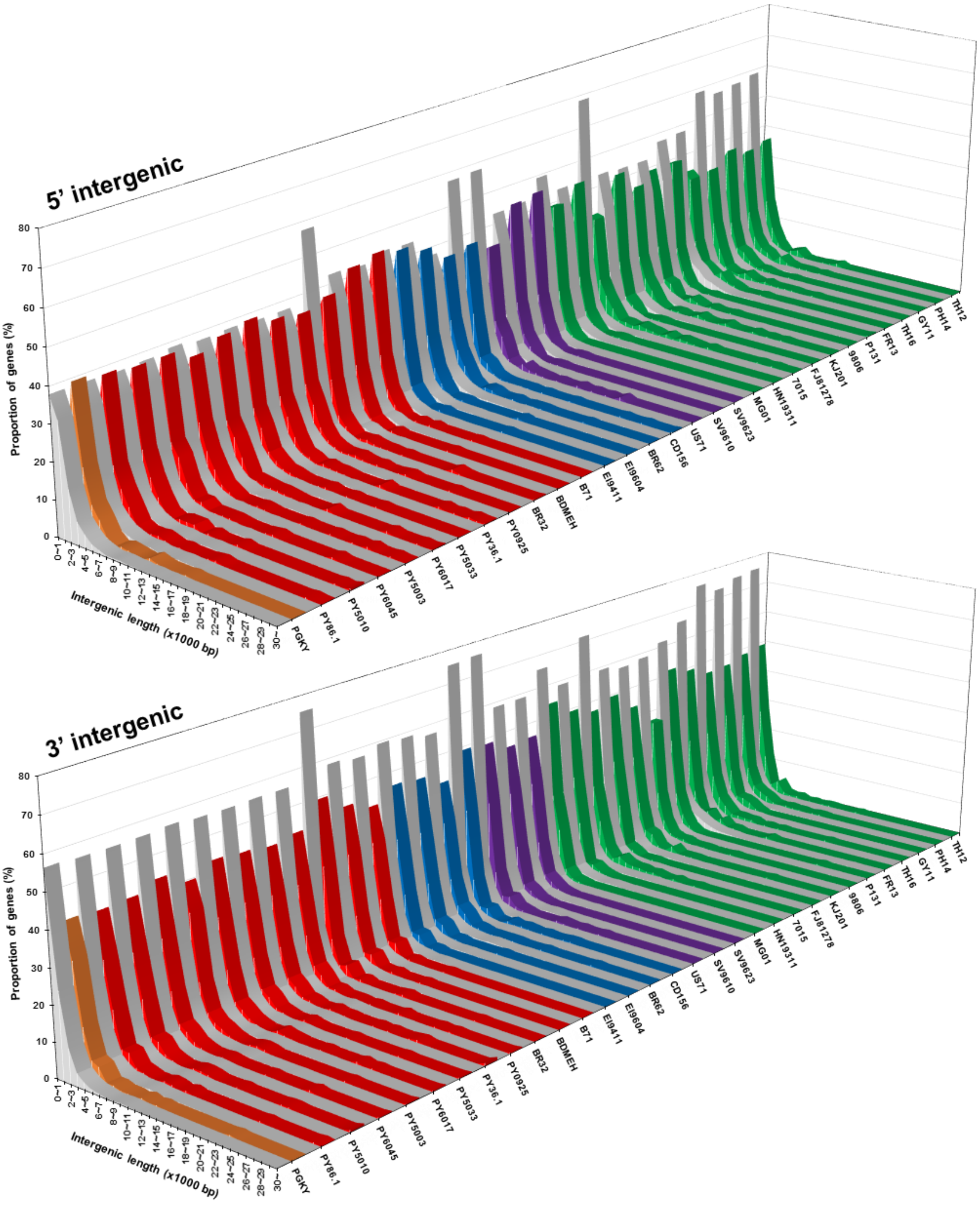


**Supplementary Figure 7. Comparison of the intergenic lengths between the *EFC* and *SCO* genes among *M. oryzae* isolates**

Results from individual pathotypes are color-coded: MoO (green), MoT (red), MoL (orange), MoE (blue) and MoS (purple). The grey histograms represent results for the *SCO* genes.

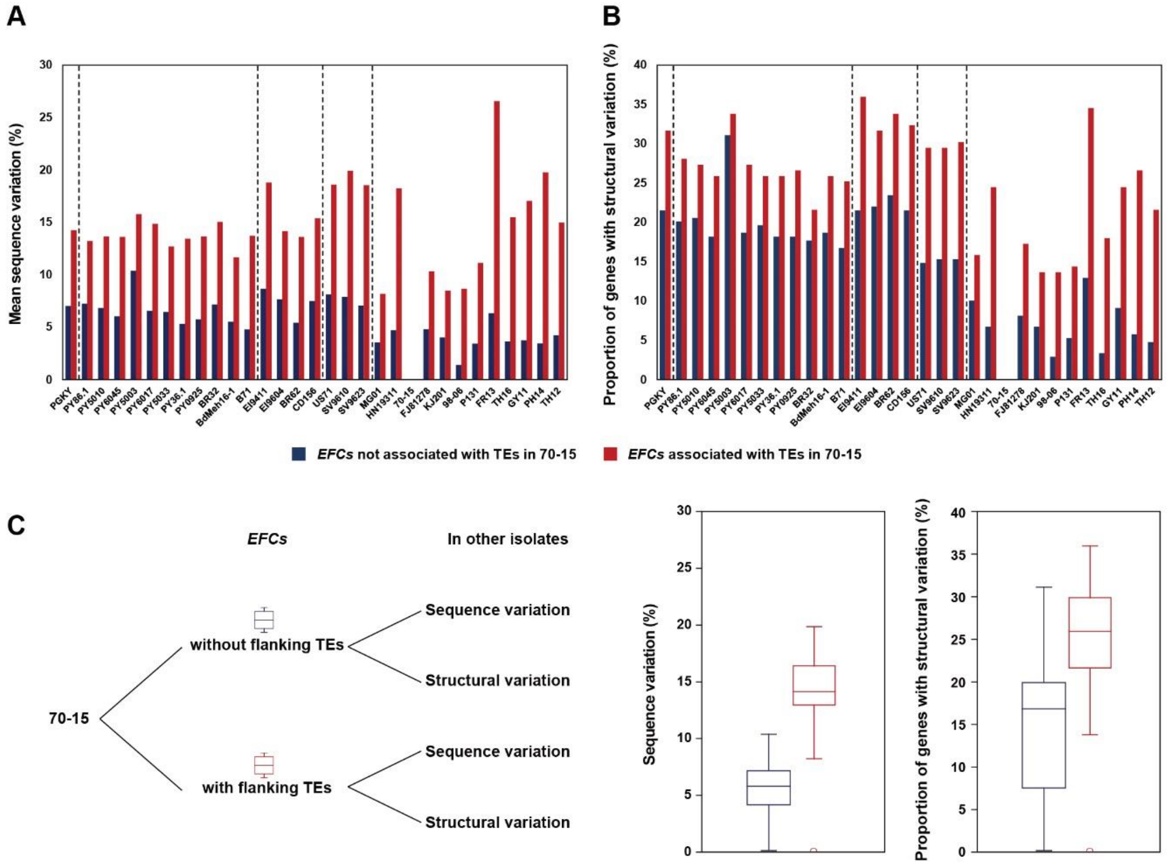


**Supplementary Figure 8. Analysis of the degree of sequence variation among the *EFC* genes and the proportion of genes with structural variation after dividing them into two groups based on the presence and absence of flanking TEs in 70-15**

The *EFC* genes in all isolates were divided into two groups depending on whether they are flanked by TEs (red) or not (cyan) in 70-15. **(A)** Average sequence variation (%) and **(B)** average proportion of *EFCs* with structural variation in two groups are shown. **(C)** The degree of sequence variation and the proportion of genes with structural variation was also compared between the groups. The boxplots show the distribution of sequence variation (left) and the proportion of genes with structural variation (right). The sequence variation was calculated using 100% - the mean sequence identity of the *EFC* genes in each isolate (%).

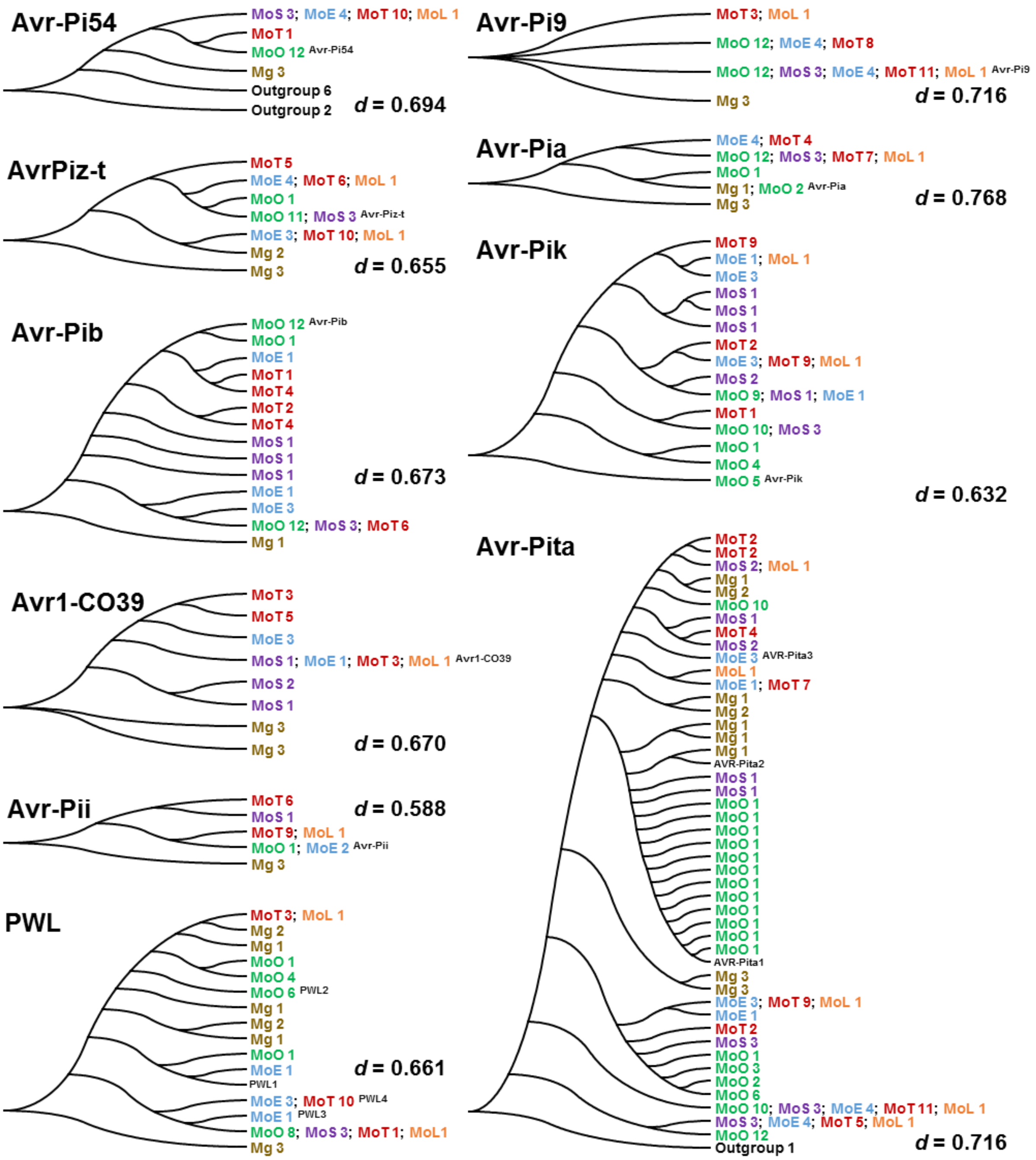


**Supplementary Figure 9. Simplified phylogenetic trees constructed using the known *AVR* genes in *Magnaporthe* isolates and the degree of congruence to the phylogenomic tree**

Individual pathotypes of *M. oryzae* and *M. grisea* isolates from *Digitaia* (labeled as Mg) are color-coded, and the number of isolates in each clade is noted. The outgroup is Magnaporthaceae species. The strict distance (*d*) indicates the degree of similarity between each *AVR* gene tree and the phylogenomics tree. The d-value < 0.5 indicates congruity between the trees and 0.5 <= *d* <=1 indicates incongruity.

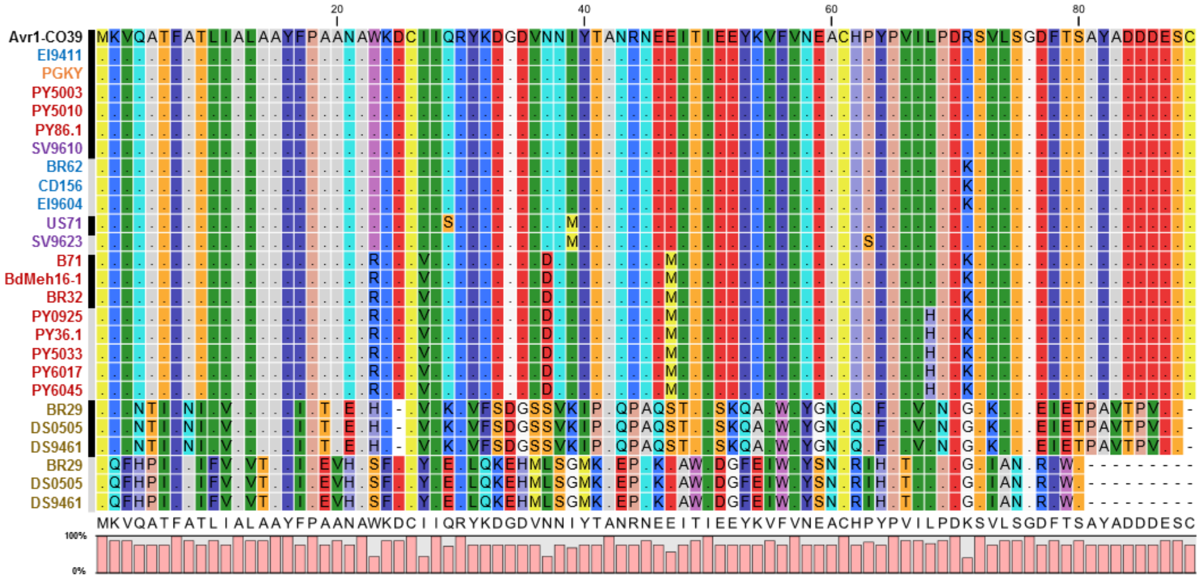


**Supplementary Figure 10. Protein sequence alignment of Avr1-Co39 encoded by *Magnaporthe* isolates**

The *Avr1-Co39* gene was initially cloned from a laboratory strain 4091-5-8, which originated from K76-79, a weeping lovegrass infecting *M. oryzae* strain (Farman et al., 2002). This AVR protein is known to be directly recognized by the rice resistance protein pair RGA4/RGA5 in the host cytoplasm (Cesari et al., 2013; Ribot et al., 2013). Although their protein sequences are different, this AVR protein has a similar three-dimensional structure to that of another *M. oryzae* AVR protein AVR-Pia (de Guillen et al., 2015). A single intact copy of *Avr1-Co39* was identified in all *M. oryzae* clades, except MoO isolates, with a few amino acid differences in certain positions, suggesting that this gene was lost in rice infecting isolates. Our results are consistent with results from previous studies on the distribution of *Avr1-Co39* in *Magnaporthe* isolates (Farman et al., 2002; Tosa et al., 2005). Each *M. grisea* isolate had two fragmented copies exhibiting the protein sequence identity of 30 % and 38 %. For example, in *M. grisea* isolate BR29, the proteins were annotated as "M_BR29_EuGene_00119491" and "M_BR29_EuGene_00119511", respectively.

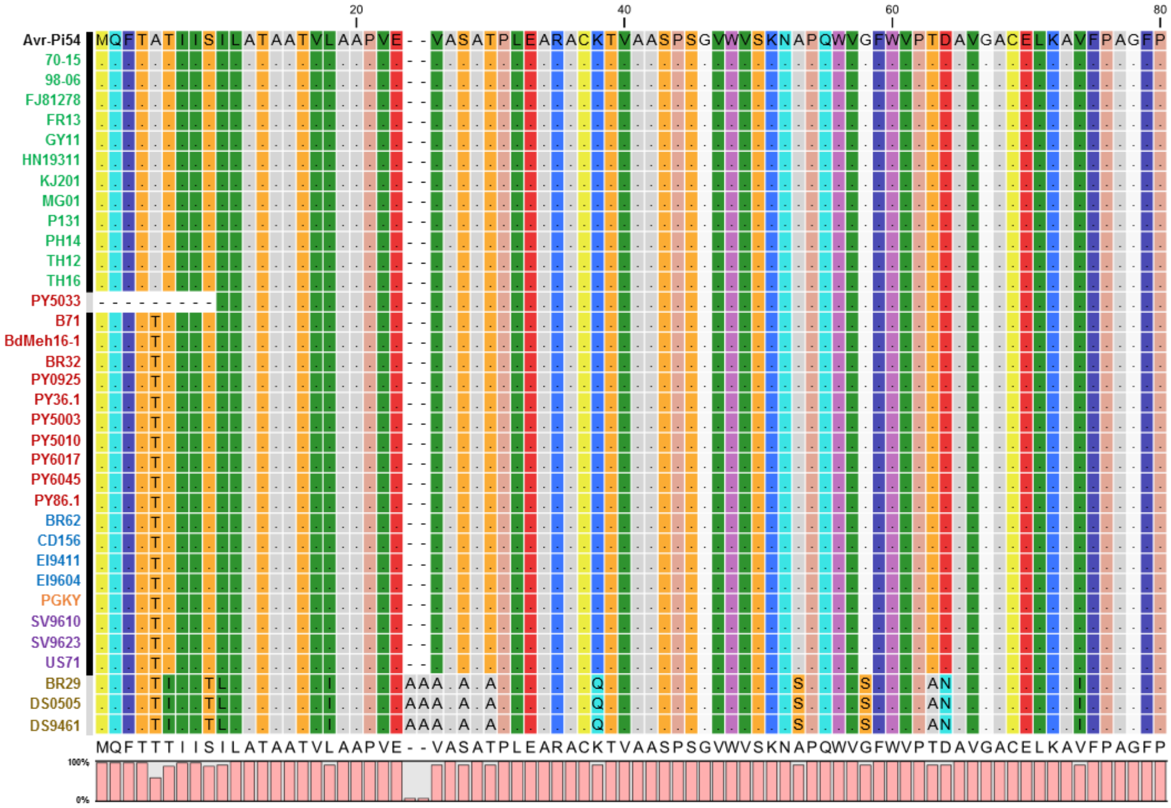


**Supplementary Figure 11. Protein sequence alignment of Avr-Pi54 encoded by *Magnaporthe* isolates**

The *Avr-Pi54* gene was first cloned from *M. oryzae* isolate RML-29, and the interaction of its product with the product of the rice *R*-gene *Pi54* was demonstrated via a yeast two-hybrid (Y2H) method. The co-expression of *Avr-Pi54* and *Pi54* in tobacco resulted in a strong hypersensitive response (Rayi et al., 2016). The figure shows that Avr-Pi54 is possibly divided into two haplotypes among *M. oryzae* pathotypes, MoO type and the rest of pathotypes, distinguished by A/T changes in the fifth position in the signal peptide region. An intact copy of the gene was also found in *M. grisea* isolates, and their gene product contains the well conserved C-terminus, and even other Magnaporthaceae species encode a protein with sequence identity to Avr-Pi54 from 40 % to 51 % (not shown).

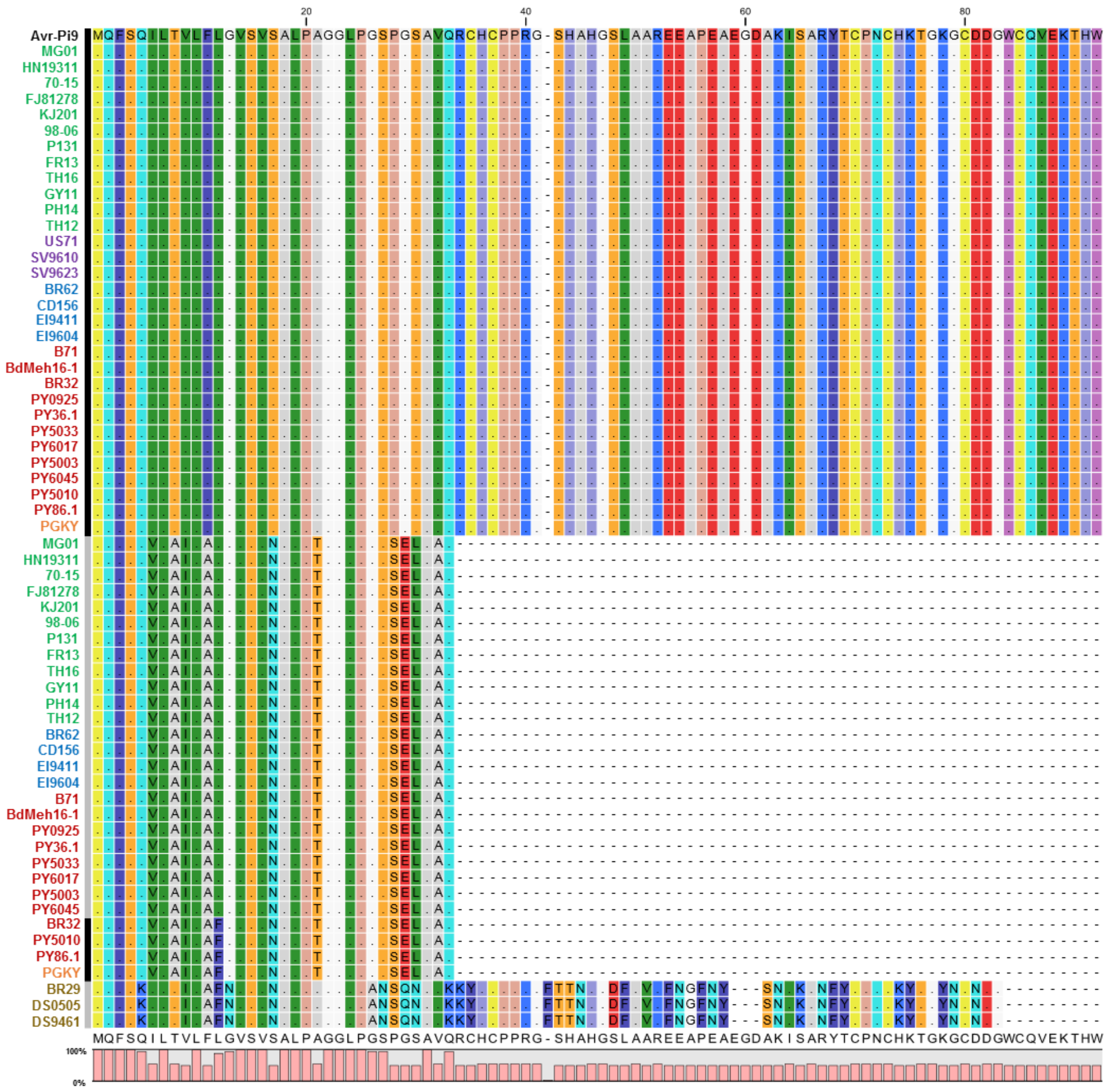

**Supplementary Figure 12. Protein sequence alignment of Avr-Pi9 encoded by *Magnaporthe* isolates**

The *Avr-Pi9* gene was first identified in *M. oryzae* isolate R88-002, and its paralog was located also found (Wu et al., 2015). An intact copy of *Avr-Pi9* without any sequence changes was found in all *M. oryzae* isolates. The *M. grisea* isolates also carry an intact gene that encodes a protein displaying 46 % sequence identity to Avr-Pi9. We also found a copy of fragmented *Avr-Pi9* paralog in all *M. oryzae* isolates, except the MoS isolates. This paralog encodes a N-terminal of protein with 24 % sequence identity to Avr-Pi9.

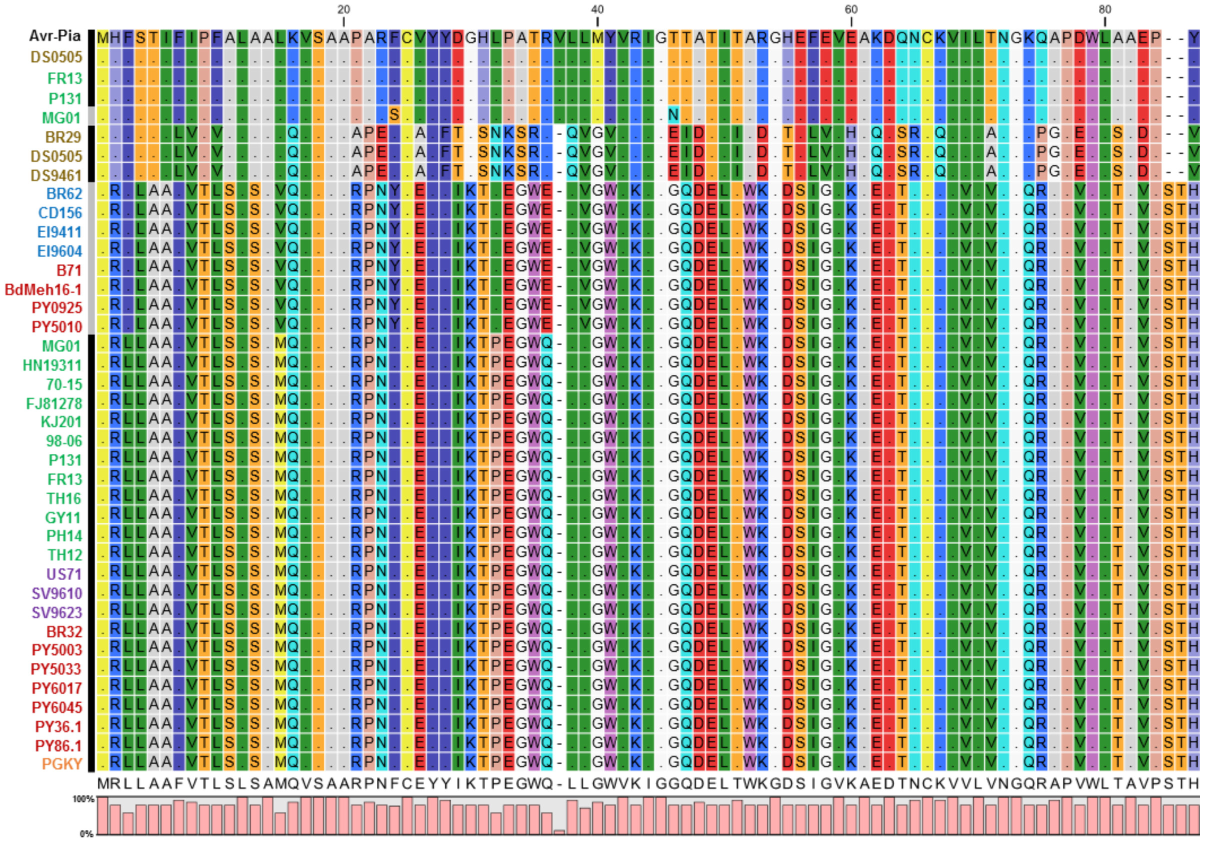

**Supplementary Figure 13. Protein sequence alignment of Avr-Pia encoded by *Magnaporthe* isolates**

The *Avr-Pia* gene was originally cloned from *M. oryzae* field isolate Ina168 via an association genetic analysis (Miki et al., 2009; Yoshida et al., 2009). The structure of Avr-Pia is similar to *M. oryzae* AVR proteins AvrPiz-t and Avr1-CO39 and ToxB, a host specific toxin encoded by wheat pathogen *Pyrenophora tritici-repentis* (Ose et al., 2015). Probably due to its similar structure with that of Avr1-CO39, the *Avr-Pia* gene product is also recognized by the rice resistance protein pair RGA4/RGA5 (Cesari et al. 2013). This gene has been found to be deleted in some strains, which appears to have been caused by a homologous recombination between transposable elements (Sone et al., 2013). We found a gene that encodes a protein similar to the Avr-Pia protein in all *M. oryzae* and *M. grisea* isolates with 41 % and 53 % sequence identity, respectively. We also found a gene with its gene product being highjly similar to the Avr-Pia protein (identity over 98 %) in *M. grisea* DS0505 and MoO isolates MG01, P131 and FR13. This result indicates that the *Avr-Pia* gene is absent in most isolates probably due to deletion.

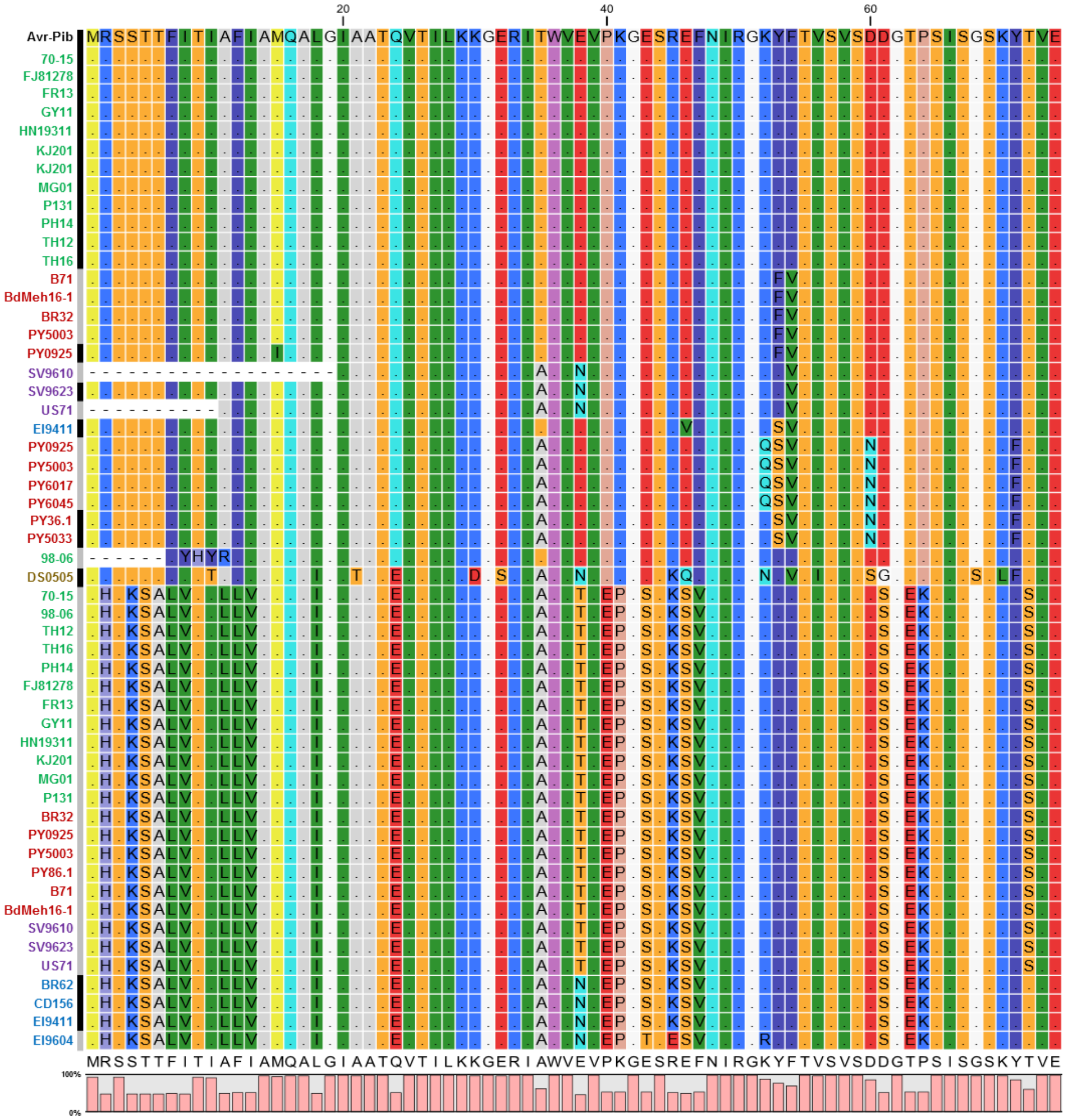


**Supplementary Figure 14. Protein sequence alignment of Avr-Pib encoded by *Magnaporthe* isolates**

The *Avr-Pib* gene was originally cloned from *M. oryzae* isolate CHL42 via map-based cloning (Zhang et al., 2015). It encodes a protein of 74 amino acids that contains a signal peptide. In most *M. oryzae* isolates, one to three copies of *Avr-Pib* were identified regardless of pathotypes. They are absent in MoL isolate PGKY and MoT isolate PY5010. The sequence identities between the reference Avr-Pib and its homologs ranged from 69 to 100 %. In *M. grisea*, only DS0505 had one intact copy of Avr-Pib with 76% sequence identity compared to the reference.

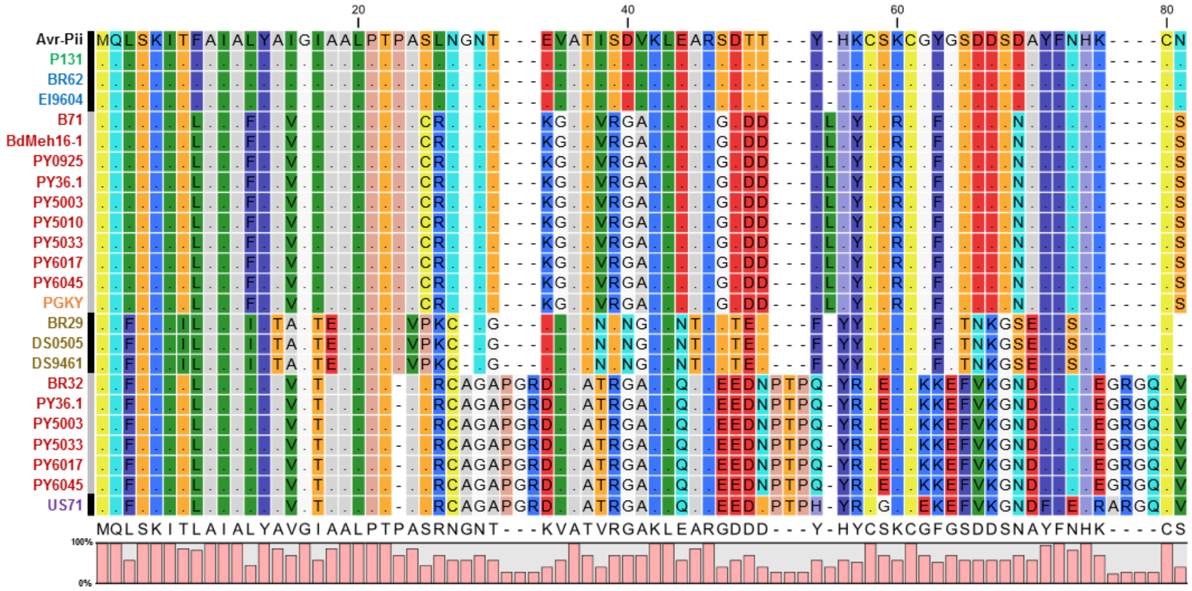


**Supplementary Figure 15. Protein sequence alignment of Avr-Pii encoded by *Magnaporthe* isolates**

The *Avr-Pii* gene was first found in *M. oryzae* isolate Ina168 along with *Avr-Pia* and *Avr-Pik* and appears to have gone thorugh both the gene gain and loss (Yoshida et al. 2009). A gene encoding a protein identical to Avr-Pii was only observed in MoS isolates BR62 and EI9604 and MoO isolate P131. The gene is absent in other MoO isolates and MoE isolates CD156 and EI9411. The MoT isolates, except PY86.1, and MoL isolates carry one to two intact copies with their products exhibiting 43 ~ 72% sequence identity to Avr-Pii. Among the MoS isolates, US71 was the only one with a gene encoding a protein that displayed 37 % sequence identity to Avr-Pii. All three *M. grisea* isolates contain an intact gene with its product exhibiting 53% identity.

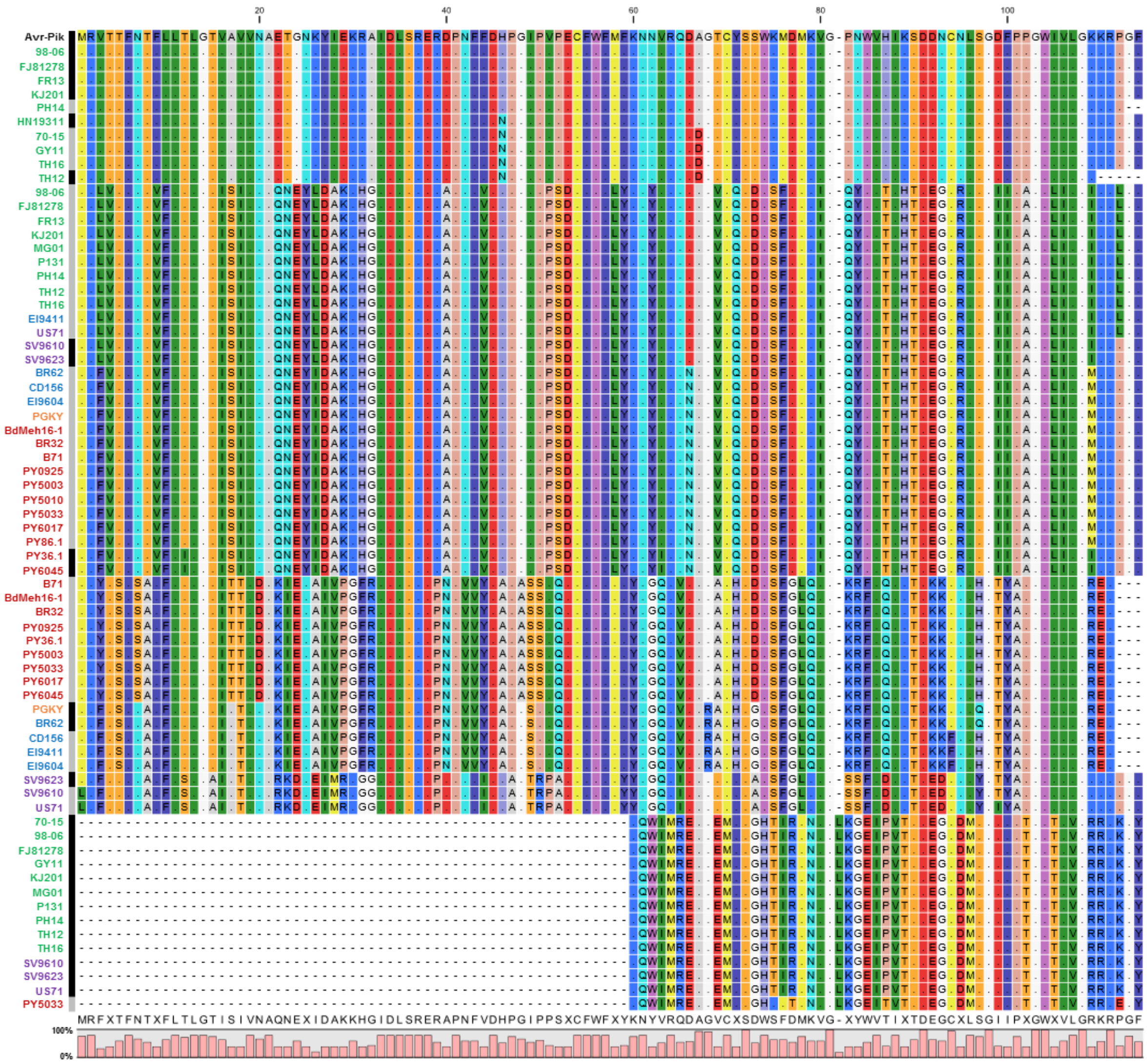

**Supplementary Figure 16. Protein sequence alignment of Avr-Pik encoded by *Magnaporthe* isolates**

The *Avr-Pik* gene was first discovered in *M. oryzae* isolate Ina168 along with *Avr-Pia* and *Avr-Pii* appears to have gone through sequence changes in addition to gene gain and loss (Yoshida et al. 2009). We found the *Avr-Pik* gene only in *M.* oryzae. This *M. oryzae*-specific gene is widely conserved across all pathotypes of *M. oryzae* with one to two copies. The sequence identity of its homolog encoded by other isolates ranged from 57 % (MoT and MoL) to 100% (MoO). Many fragmented sequences were also observed in all isolates. The MoT, MoL and MoE isolates have an extra gene, which encodes a protein resembling N-terminus of Avr-Pik and MoO and MoS isolates have an extra gene, which encodes a protein resembling C-terminus of Avr-Pik.

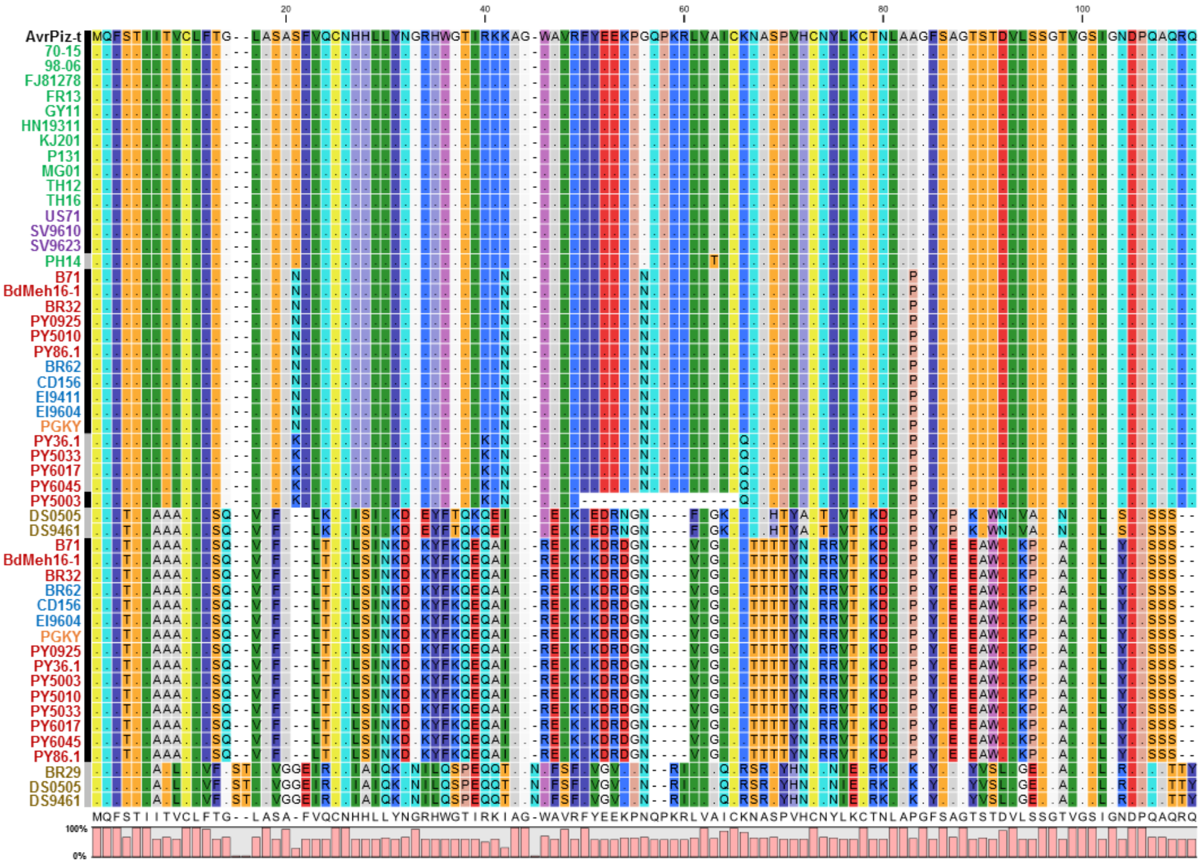

**Supplementary Figure 17. Protein sequence alignment of AvrPiz-t encoded by *Magnaporthe* isolates**

The avirulence function of AvrPiz-t was first identified using *M. oryzae* isolate 81278ZB15, and the distruption of the gene by an insertion of repetitive element caused the loss of its avirulence function (Li et al., 2009). Various haplotypes of Avr-Pizt were identified using 711 isolates from 38 countries and this gene is associated with transposable elements (Chen et al., 2014; Sirisathaworn et al., 2017). We found that AvrPiz-t was well conserved among all *M. oryzae* isolates (sequence identity from 94 % of MoT isolates to 100% of MoO isolates). The MoL, MoT and MoE isolates carry an additional intact copy of its homolog. The protein encoded by the homolog displayed relatively low sequence identity (35%) to AvrPiz-t. This homolog was not detected in the MoS and MoO isolates. *M. grisea* isolates contain a gene that encodes a protein exhibiting 39~41 % identity to AvrPiz-t.

A

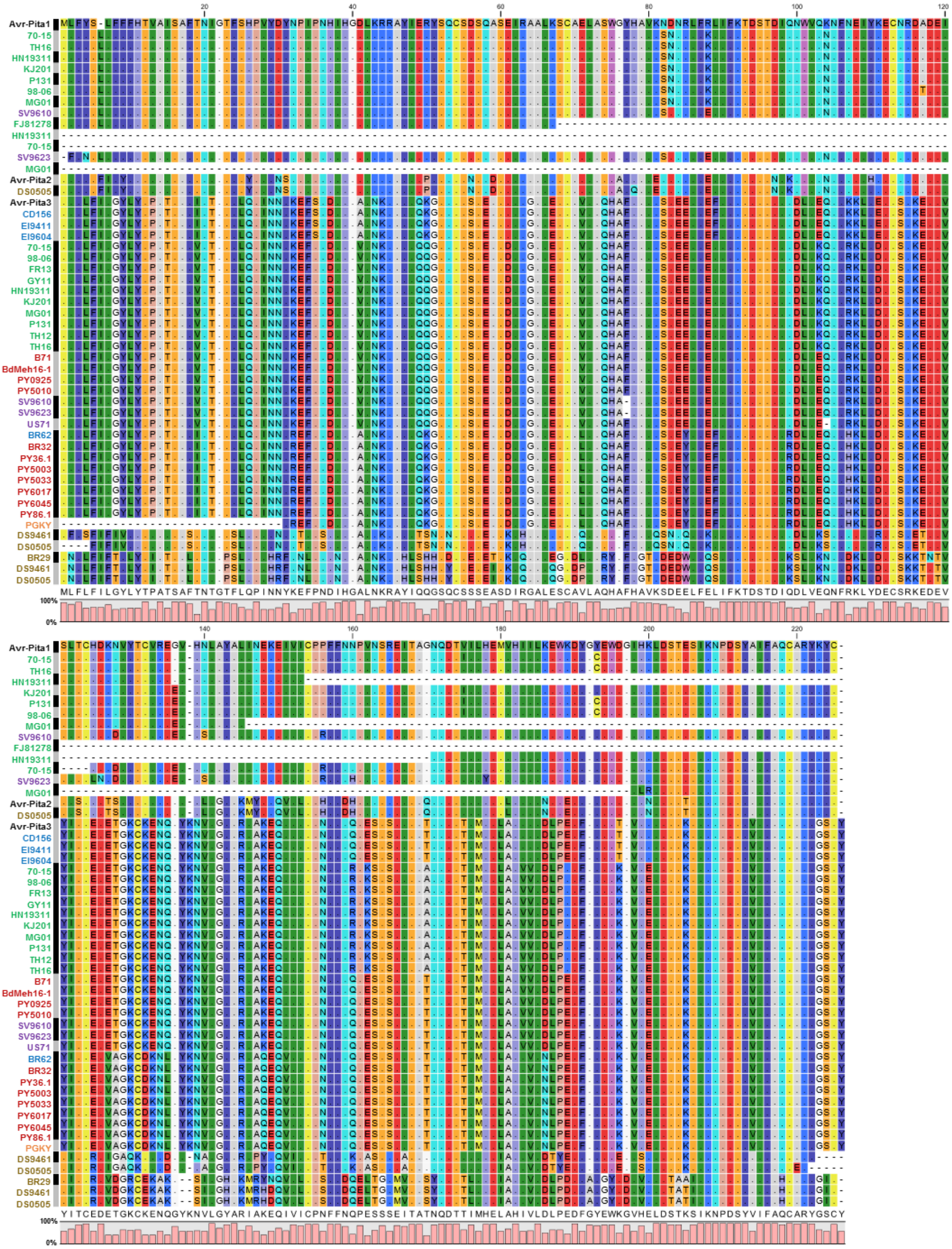
Avr-Pita1
70-15
TH16
HN19311
KJ201
P131
98-06
MG01
SV9610
FJ81278
HN19311
70-15
SV9623
MG01
Avr-Pita2
DS0505
Avr-Pita3
CD156
EI9411
EI9604
70-15
98-06
FR13
GY11
HN19311
KJ201
MG01
P131
TH12
TH16
B71
BdMeh16-1
PY0925
PY5010
SV9610
SV9623
US71
BR62
BR32
PY36.1
PY5003
PY5033
PY6017
PY6045
PY86.1
PGKY
DS9461
DS0505
BR29
DS9461
DS0505
100%
0%

**B**

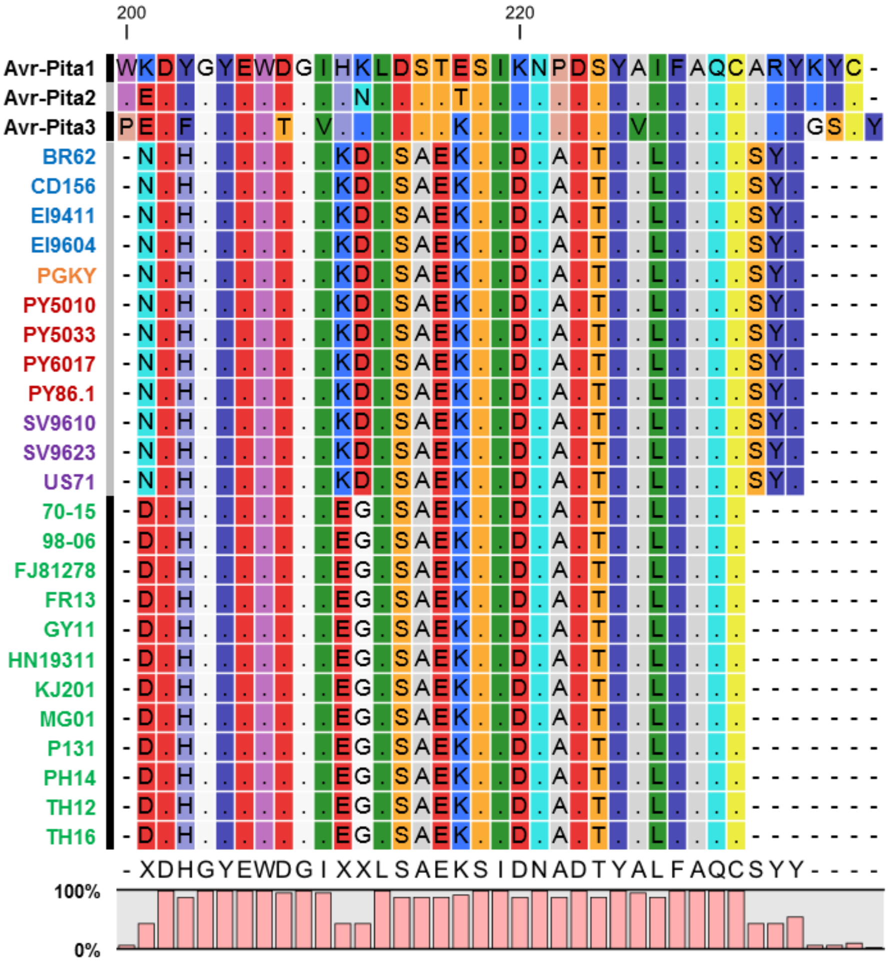

200
220
Avr-Pita1
Avr-Pita2
Avr-Pita3
BR62
CD156
EI9411
EI9604
PGKY
PY5010
PY5033
PY6017
PY86.1
SV9610
SV9623
US71
70-15
98-06
FJ81278
FR13
GY11
HN19311
KJ201
MG01
P131
PH14
TH12
TH16
- XDHGYEWDGIXXLSAEKSIDNADTYALFAQCSYY----
100%
0%

C

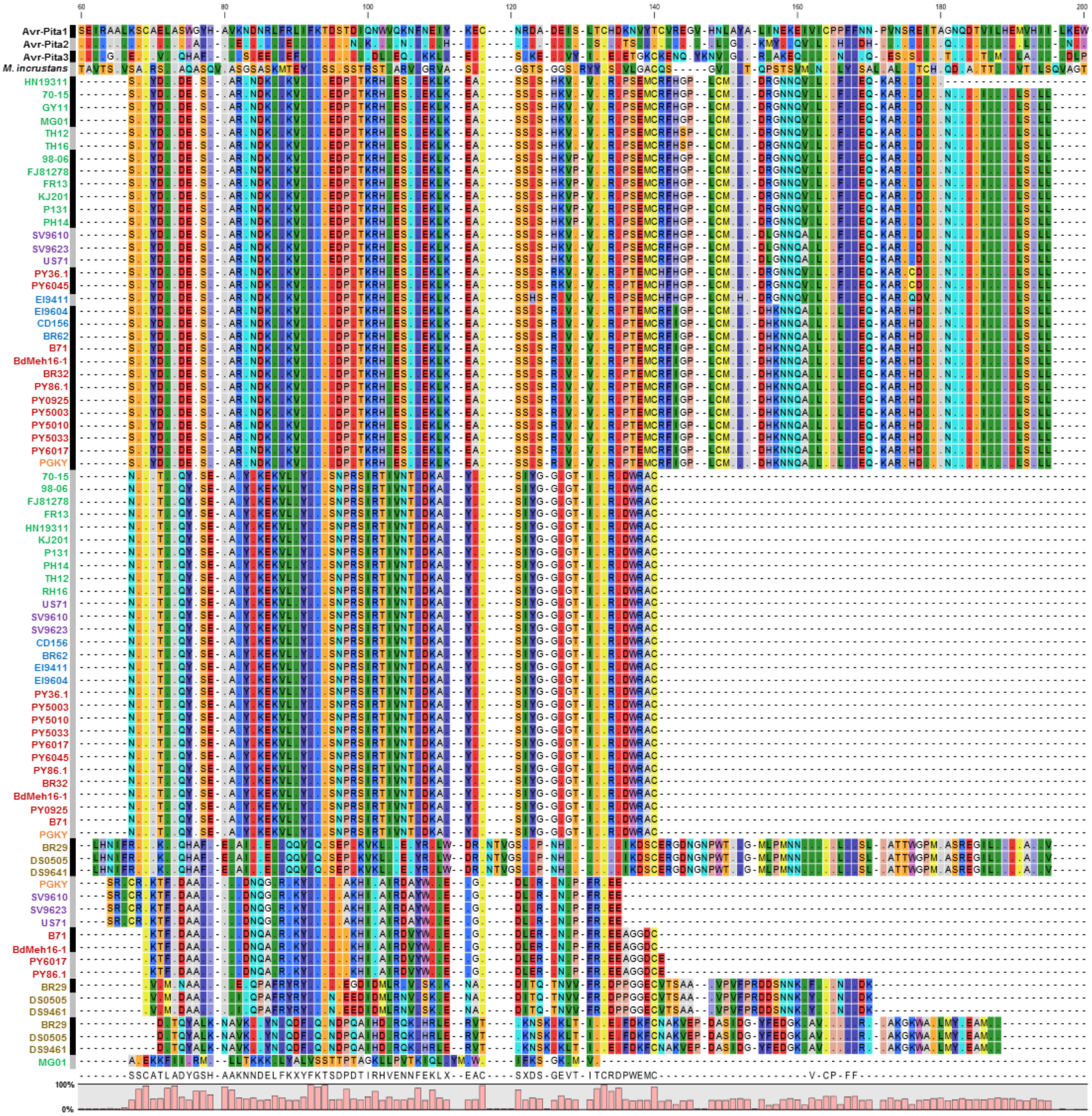

Avr-Pita1
Avr-Pita2
Avr-Pita3
M. incrustans
HN19311
70-15
GY11
MG01
TH12
TH16
98-06
FJ81278
FR13
KJ201
P131
PH14
SV9610
SV9623
US71
PY36.1
PY6045
EI9411
EI9604
CD156
BR62
B71
BdMeh16-1
BR32
PY86.1
PY0925
PY5003
PY5010
PY5033
PY6017
PGKY
BR29
DS0505
DS9641
DS9461
100%
0%

**Supplementary Figure 18. Sequence alignment of the *Avr-Pita* gene family products encoded by *Magnaporthe* isolates**

The evolution and variation of the *Avr-Pita* gene family has been well studied. Members of the family appear highly variable, and their gene products seem to function as a metalloprotease (Jia et al., 2000). Members of the family are located multiple chromosomes (Chuma et al., 2011). Their chromosomal translocations were mediated by retrotransposons (Chuma et al., 2011), and a transposon such as POT3 was inserted into the promoter of the gene in some isolates, causing the loss of avirulence function (Kang et al., 2001; Zhou et al., 2007). The transposons also seem to have mediated rapid sequence diversification within the family (Singh et al., 2014). The *Avr-Pita* family consists of Avr-Pita1, Avr-Pita2 and Avr-Pita3. A previous study reported that Avr-Pita1 and Avr-Pita2 were present in both *M. oryzae* and *M. grisea*, and Avr-Pita3 was present only in *M. oryzae* (Khang et al., 2008). **(A)** In our analysis, *Avr-Pita1* was detected only in MoO and MoS isolates, *Avr-Pita2* was detected only in *M. grisea* isolate DS0505, and *Avr-Pita3* was detected in all *M. oryzae* pathotypes. *M. grisea* isolates also contain a gene that encode a protein with similar sequences to Avr-Pita3. **(B)** and **(C)** Partial sequences were sporadically detected in both *M. oryzae* and *M. grisea* isolates. Rice isolate MG01 has an intact *Avr-Pita* gene and five partial sequences. **(C)** Unlike other *AVRs*, a partial sequence was detected in *M. incrustans* with 44.4% identity at the protein level. Although this partial sequence does not contain any introns, its product aligned with all four exons of *Avr-Pita* encoded by Mo 70-15 and Mg DS0505. The partial sequence was predicted to be the metallopeptidase catalytic domain (IPR024079) and is a part of the first exon of the gene MIG_09721-R0. The overall pattern suggested that the *Avr-Pita* gene(s) existed in Magnaporthaceae species before the speciation of *Magnaporthe,* and that they have been diversified after gaining the introns.

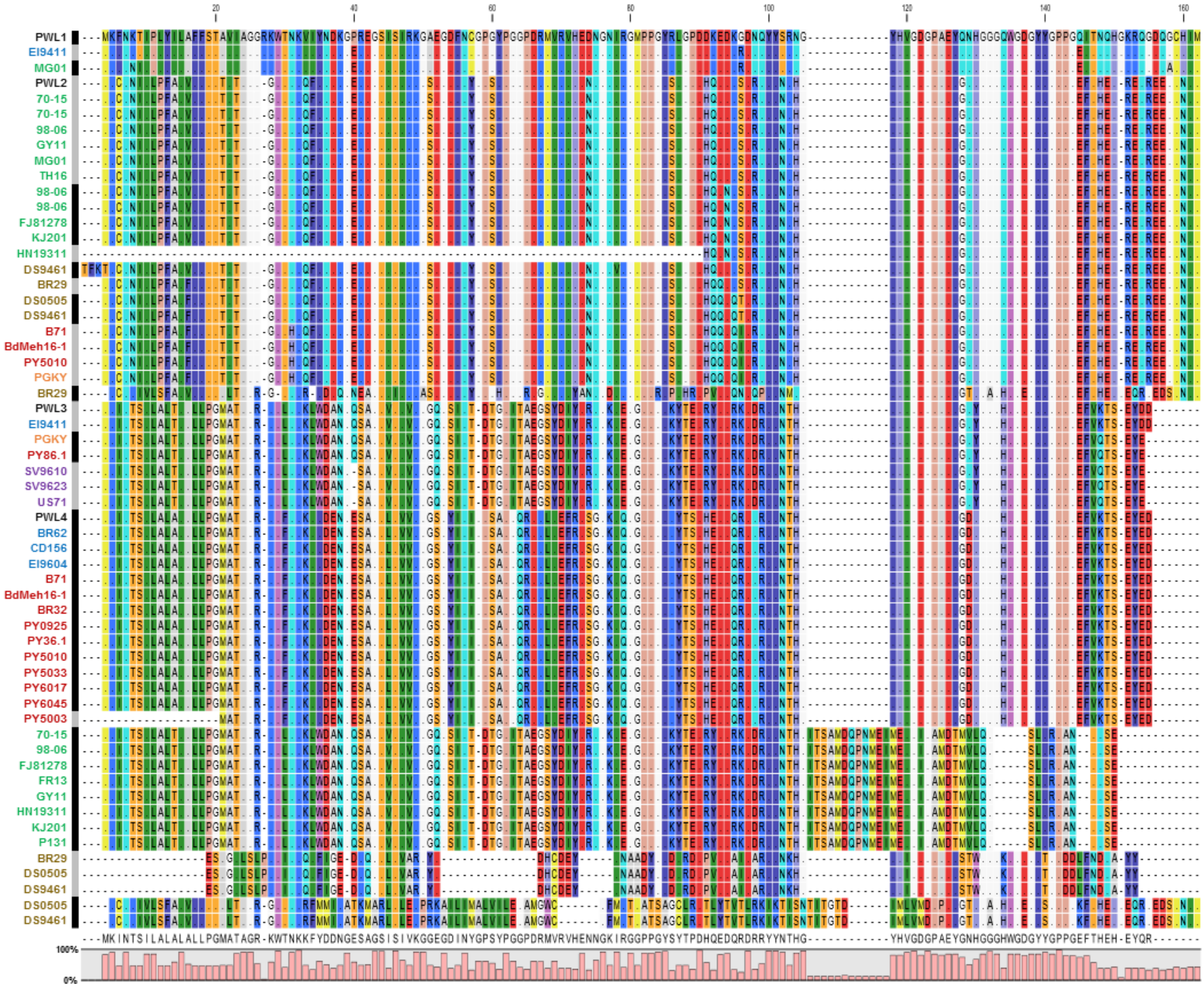


**Supplementary Figure 19. Sequence alignment of members of the PWL gene family encoded by *Magnaporthe* isolates**

Some members of this host specificity gene family prevent the fungus from infecting weeping lovegrass (Kang et al., 1995). This family consists of two paralogous forms *PWL1*/*PWL2* and *PWL3*/*PWL4*. This is a genus-specific gene family probably originated from a common ancestor of *M. oryzae* and *M. grisea*. A complete gene and partial sequences of *PWL2* were found in *M. grisea*, but only a partial sequence of *PWL3* was found in *M. grisea*. In *M. oryzae*, some rice isolates had multiple copies of *PWL2* or had none, but *PWL3* was presented as either partial sequence or absent. *PWL2* was absent in MoS e isolates, but a partial sequence of *PWL3* was remained in them. In contrast, an intact PWL3 was predicted in monophyletic MoE and MoT isolates except PY86.3, which was grouped with MoL pathotype isolate PGKY. In this monophyletic group, *PWL2* was lost in most isolates.

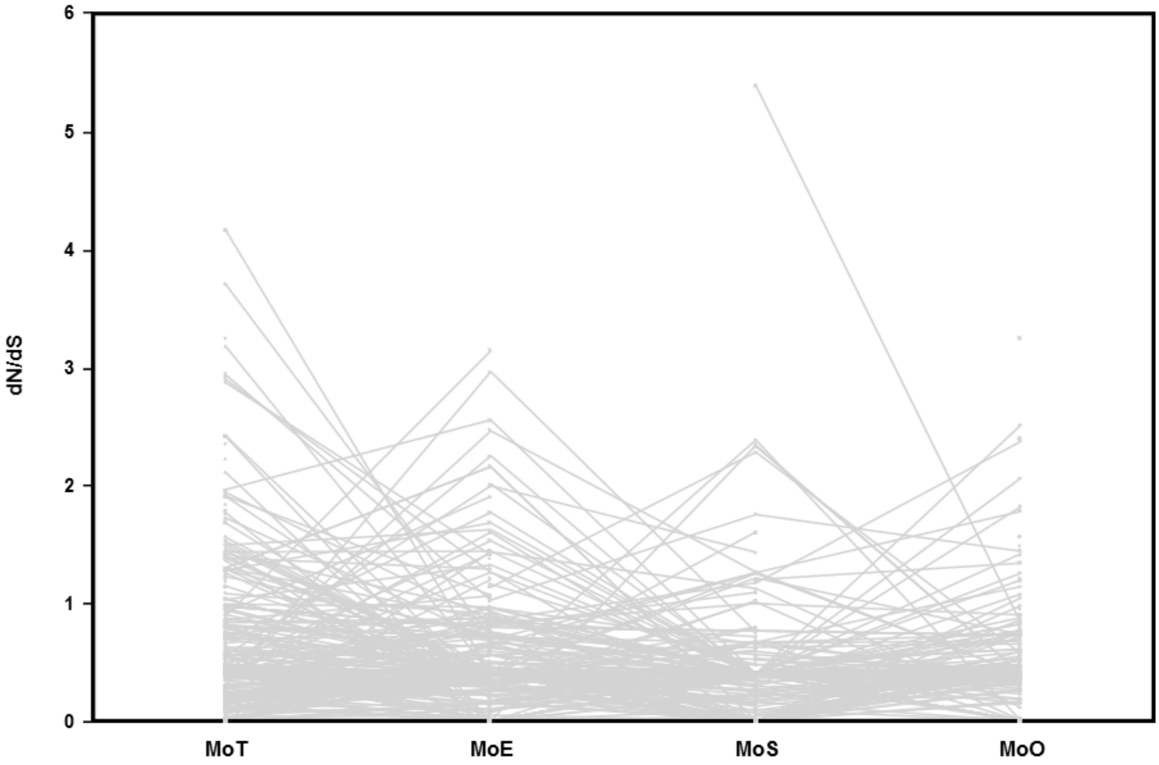


**Supplementary Figure 20. Selection pressure on the *EFC* genes in four *M. oryzae* pathotypes**

The ω (dN/dS) ratio for each *EFC* gene sequence in the isolates of these pathotypes is presented as a line graph. The broken lines are caused because we discarded the genes with the ratio of > 10.

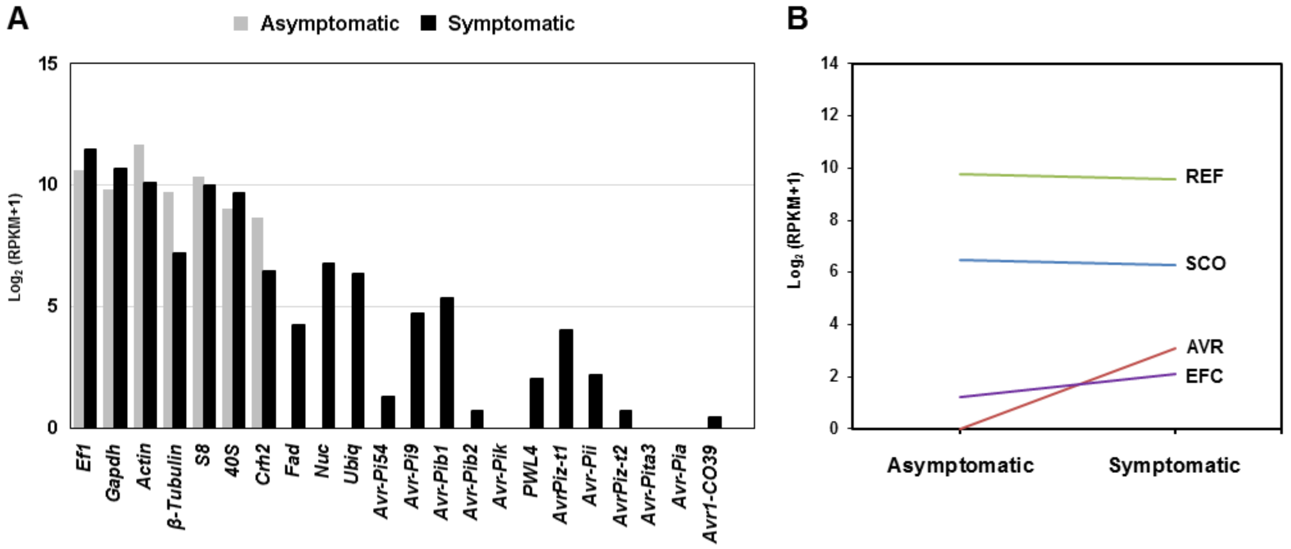


**Supplementary Figure 21. Expression patterns of *REFs*, *AVRs*, *EFCs* and *SCOs* during infection of wheat by MoT isolate BR32**

**(A)** Expression levels of *REFs* and *AVRs* in asymptomatic (grey) and symptomatic (black) wheat plant samples are shown. **(B)** Averaged expression levels of the four gene sets in two stages are shown.

**References**


Cesari, S., Thilliez, G., Ribot, C., Chalvon, V., Michel, C., Jauneau, A., et al. (2013). The rice resistance protein pair RGA4/RGA5 recognizes the *Magnaporthe oryzae* effectors AVR-Pia and AVR1-CO39 by direct binding. *Plant Cell* 25, 1463-1481. doi: 10.1105/tpc.112.107201

Chen, C., Chen, M., Hu, J., Zhang, W., Zhong, Z., Jia, Y., et al. (2014). Sequence variation and recognition specificity of the avirulence gene *AvrPiz-T* in *Magnaporthe oryzae* field populations. *Fungal Genom. Biol.* 4:1. doi: 10.4172/2165-8056.1000113

Chuma, I., Isobe, C., Hotta, Y., Ibaragi, K., Futamata, N., Kusaba, M., et al. (2011). Multiple translocation of the *AVR-Pita* effector gene among chromosomes of the rice blast fungus *Magnaporthe oryzae* and related species. *PLoS Pathog.* 7:e1002147. doi: 10.1371/journal.ppat.1002147

De Guillen, K., Ortiz-Vallejo, D., Gracy, J., Fournier, E., Kroj, T., and Padilla, A. (2015). Structure analysis uncovers a highly diverse but structurally conserved effector family in phytopathogenic fungi. *PLoS Pathog.* 11:e1005228. doi: 10.1371/journal.ppat.1005228

Farman, M. L., Eto, Y., Nakao, T., Tosa, Y., Nakayashiki, H., Mayama, S., et al. (2002). Analysis of the structure of the *AVR1-CO39* avirulence locus in virulent rice-infecting isolates of *Magnaporthe grisea*. *Mol. Plant-Microbe Interact.* 15, 6-16. doi: 10.1094/MPMI.2002.15.1.6

Jia, Y., McAdams, S. A., Bryan, G. T., Hershey, H. P., and Valent, B. (2000). Direct interaction of resistance gene and avirulence gene products confers rice blast resistance. *EMBO J.* 19, 4004-4014. doi: 10.1093/emboj/19.15.4004

Kang, S., Lebrun, M. H., Farrall, L., and Valent, B. (2001). Gain of virulence caused by insertion of a Pot3 transposon in a *Magnaporthe grisea* avirulence gene. *Mol. Plant-Microbe Interact* 14, 671-674. doi: 10.1094/MPMI.2001.14.5.671

Kang, S., Sweigard, J. A., and Valent, B. (1995). The *PWL* host specificity gene family in the blast fungus *Magnaporthe grisea*. *Mol. Plant-Microbe Interact.* 8, 939-948. doi: 10.1094/MPMI-8-0939

Khang, C. H., Park, S. Y., Lee, Y. H., Valent, B., and Kang, S. (2008). Genome organization and evolution of the *AVR-Pita* avirulence gene family in the *Magnaporthe grisea* species complex. *Mol. Plant-Microbe Interact.* 21, 658-670. doi: 10.1094/MPMI-21-5-0658

Li, W., Wang, B. H., Wu, J., Lu, G. D., Hu, Y. J., Zhang, X., et al. (2009). The *Magnaporthe oryzae* avirulence gene *AvrPiz-t* encodes a predicted secreted protein that triggers the immunity in rice mediated by the blast resistance gene *Piz-t*. *Mol. Plant-Microbe Interact.* 22, 411-420. doi: 10.1094/MPMI-22-4-0411

Miki, S., Matsui, K., Kito, H., Otsuka, K., Ashizawa, T., Yasuda, N., et al. (2009). Molecular cloning and characterization of the *AVR-Pia* locus from a Japanese field isolate of *Magnaporthe oryzae*. *Mol. Plant Pathol.* 10, 361-374. doi: 10.1111/j.1364-3703.2009.00534.x

Ose, T., Oikawa, A., Nakamura, Y., Maenaka, K., Higuchi, Y., Satoh, Y., et al. (2015). Solution structure of an avirulence protein, AVR-Pia, from *Magnaporthe oryzae*. *J. Biomol. NMR* 63, 229-235. doi: 10.1007/s10858-015-9979-7

Ray, S., Singh, P. K., Gupta, D. K., Mahato, A. K., Sarkar, C., Rathour, R., et al. (2016). Analysis of *Magnaporthe oryzae* genome reveals a fungal effector, which is able to induce resistance response in transgenic rice line containing resistance Gene, *Pi54*.

*Front. Plant Sci.* 7:1130. doi: 10.3389/fpls.2016.01140

Ribot, C., Cesari, S., Abidi, I., Chalvon, V., Bournaud, C., Vallet, J., et al. (2013). The *Magnaporthe oryzae* effector AVR1CO39 is translocated into rice cells independently of a fungal-derived machinery. *Plant J.* 74, 1-12. doi: 10.1111/tpj.12099

Singh, P. K., Thakur, S., Rathour, R., Variar, M., Prashanthi, S. K., Singh, A. K., et al. (2014). Transposon-based high sequence diversity in *Avr-Pita* alleles increases the potential for pathogenicity of *Magnaporthe oryzae* populations. *Funct. Integrat. Genomics* 14, 419-429. doi: 10.1007/s10142-014-0369-0

Sirisathaworn, T., Srirat, T., Longya, A., and Jantasuriyarat, C. J. A.(2017). Evaluation of mating type distribution and genetic diversity of three *Magnaporthe oryzae* avirulence genes, *PWL-2*, *AVR-Pii* and *Avr-Piz-t*, in Thailand rice blast isolates. *ANRES* 51, 7-14. doi: 10.1016/j.anres.2016.08.005

Sone, T., Takeuchi, S., Miki, S., Satoh, Y., Ohtsuka, K., Abe, A., et al. (2013). Homologous recombination causes the spontaneous deletion of *AVR-Pia* in *Magnaporthe oryzae*. *FEMS Microbiol. Lett.* 339, 102-109. doi: 10.1111/1574-6968.12058

Tosa, Y., Osue, J., Eto, Y., Oh, H. S., Nakayashiki, H., Mayama, S., et al. (2005). Evolution of an avirulence gene, *AVR1-CO39*, concomitant with the evolution and differentiation of *Magnaporthe oryzae*. *Mol. Plant-Microbe Interact.* 18, 1148-1160. doi: 10.1094/MPMI-18-1148

Wu, J., Kou, Y. J., Bao, J. D., Li, Y., Tang, M. Z., Zhu, X. L., et al. (2015). Comparative genomics identifies the *Magnaporthe oryzae* avirulence effector *AvrPi9* that triggers *Pi9*-mediated blast resistance in rice. *New Phytol.* 206, 1463-1475. doi: 10.1111/nph.13310

Yoshida, K., Saitoh, H., Fujisawa, S., Kanzaki, H., Matsumura, H., Yoshida, K., et al. (2009). Association genetics reveals three novel avirulence genes from the rice blast fungal pathogen *Magnaporthe oryzae*. *Plant Cell* 21, 1573-1591. doi: 10.1105/tpc.109.066324

Zhang, S. L., Wang, L., Wu, W. H., He, L. Y., Yang, X. F., and Pan, Q. H. (2015). Function and evolution of *Magnaporthe oryzae* avirulence gene *AvrPib* responding to the rice blast resistance gene *Pib*. *Sci. Rep.* 5:11642. doi: 10.1038/srep11642

Zhou, E. X., Jia, Y. L., Singh, P., Correll, J. C., and Lee, F. N. (2007). Instability of the *Magnaporthe oryzae* avirulence gene *AVR-Pita* alters virulence. *Fungal Genet. Biol.* 44, 1024-1034. doi: 10.1016/j.fgb.2007.02.003
